# Physiological and developmental disturbances caused by Botryosphaeria dieback in the annual stems of grapevine

**DOI:** 10.3389/fpls.2024.1394821

**Published:** 2024-04-23

**Authors:** Florian Moret, Lucile Jacquens, Philippe Larignon, Gilles Clément, Cindy Coppin, Elodie Noirot, Pierre-Emmanuel Courty, Florence Fontaine, Marielle Adrian, Sophie Trouvelot

**Affiliations:** ^1^ Agroécologie, INRAE, Institut Agro, Univ. Bourgogne, Dijon, France; ^2^ IFV, Pôle Rhône-Méditerranée, Rodilhan, France; ^3^ Institut Jean-Pierre Bourgin, INRAE, AgroParisTech, CNRS, Université Paris-Saclay, Versailles, France; ^4^ Université de Reims Champagne-Ardenne, Unité Résistance Induite et Bioprotection des Plantes RIBP USC INRAE 1488, Reims, France

**Keywords:** *Vitis vinifera*, grapevine trunk diseases, targeted genes, metabolomic, cytology

## Abstract

Botryosphaeria dieback is a grapevine trunk disease caused by fungi of the Botryosphaeriaceae family, which attacks more specifically the woody tissues. The infection leads to different symptoms including a severe form with a leaf drop as well as premature plant death. Botryosphaeria dieback causes major economic losses, since no effective treatment is yet available. A better understanding is necessary to find solutions to fight this disease. In this study, our objective was to characterize the “leaf drop” form by (1) looking for the presence of pathogens in the basal internodes of stems, (2) quantifying blocked vessels by tylosis and/or gummosis, and (3) describing the impact of the disease on vine physiology (gene expression and metabolome) and development (establishment and functioning of the cambium and phellogen) at the level of xylem and phloem of basal stem internodes. Our study has shown that Botryosphaeriaceae were present in both phloem and xylem of the basal internodes of the annual stem, with xylem vessels obturated. We have also clearly demonstrated that gene expression and metabolite profiles were strongly modified in both xylem and phloem of diseased plants. Differences in stems between healthy (control, C) and diseased (D) plants were low at flowering (vines not yet symptomatic), higher at the onset of symptom expression and still present, although less marked, at full disease expression. qRT-PCR analysis showed in both phloem and xylem an overexpression of genes involved in plant defense, and a repression of genes related to meristematic activity (i.e. vascular cambium and phellogen). Metabolomic analysis showed specific fingerprints in stems of healthy and diseased plants from the onset of symptom expression, with an increase of the level of phytoalexins and mannitol, and a decrease of 1-kestose one. At the structural level, many alterations were observed in internodes, even before the onset of symptoms: a classical obstruction of xylem vessels and, for the first time, a disorganization of the secondary phloem with an obstruction of the sieve plates by callose. The disease modifies the development of both secondary phloem (liber) and phellogen. Altogether, this study combining different approaches allowed to highlight deep vine dysfunction in the internodes at the base of stems, that may explain vine decline due to Botryosphaeria dieback.

## Introduction

Cultivated grapevine (*Vitis vinifera* L.) is an economically very important crop, covering 7.2 mha worldwide, with a grape and wine production of 79.4 mt and 262 mhl respectively, and an international wine trade (export) of about 37.6 billion euros (OIV (International Organisation of Vine and Wine), 2022). However, one major problem for winegrowers is the vine susceptibility to a wide range of fungal pathogens responsible for cryptogamic diseases. Some of them are specific of the plant aerial organs such as leaves and berries (i.e. mildews, bunch rots), others are localized into the roots (Armillaria) and, finally, some are able to attack the perennial parts of the wood. These latter correspond to a complex of xylem-inhabiting fungi responsible for grapevine trunk diseases (GTDs). GTDs are a group of various and complex diseases, which thus attack woody organs and often lead to plant death (Bertsch et al., 2013; Fontaine et al., 2016 for reviews). Although these diseases are not recent, they are considered as emerging since their incidence has increased significantly over the past decades (Bertsch et al., 2013). In this context, controlling GTDs is a significant challenge for the grape industry since they impact production, longevity of vines and more generally the economy of the production system. In Europe, some GTDs were traditionally controlled by sodium arsenite applications (Larignon, 2004; Bisson et al., 2006; Spinosi and Févotte, 2008), but its use was banned in France in 2001 regarding its carcinogenic activity (IARC (International Agency for Research on Cancer), 1987). To date, there are no effective and available control methods against GTDs and the lack of alternative strategies to fight them could exacerbate the situation.

Among GTDs, Botryosphaeria dieback, previously called black dead arm (Lehoczky, 1974) causes considerable damage in vineyards (Larignon, 2016; Guérin-Dubrana et al., 2019). Disease symptoms are characterized by stunted growth, cankers, wood necrosis, as well as cordon and cane dieback (Phillips, 1998; Van Niekerk et al., 2006; Savocchia et al., 2007; Mondello et al., 2018). In some cases, reduced bud burst has been reported (Castillo-Pando et al., 2001; Qiu et al., 2011; Wunderlich et al., 2011a). A severe form is characterized by a leaf drop associated to a poor cane maturation, a shriveling and drying of inflorescences or fruit clusters and a chronic form with leaf discoloration from the margin to the blade (Larignon et al., 2001). Typical foliar symptoms of the chronic form vary between white and red cultivars. White cultivars show yellowish-orange discolored spots on the margins of the leaves and the blade, whereas red cultivars present wine-red spots. Moreover, a cross-section in the trunk shows a yellowish-orange area at the edge of a characteristic brown stripe, limited at few millimeters in depth, where xylem vessels are clogged. The infection by the pathogens blocks xylem vessels by the formation of tylosis or gummosis, as well as by their physical presence (Bertsch et al., 2013). Finally, grey sectorial necrosis can be also observed in woody tissue (Larignon, 2012).

Botryosphaeria dieback is due to ascomycete fungi belonging to the family of Botryosphaeriaceae and capable of developing in the wood by producing necrosis or cankers (Wunderlich et al., 2011b; Dissanayake et al., 2016). They are well-known pathogens causing dieback in a wide range of plant hosts such as apples, pine trees and grapevines (Liu et al., 2012; Mehl et al., 2013). The importance and wide distribution of these fungi in different vineyards has been largely reported (Larignon et al., 2009; Bertsch et al., 2013; Yan et al., 2013). To date, more than 25 species of Botryosphaeriaceae have been associated with Botryosphaeria dieback in grapevine (Urbez-Torres, 2011; Berraf-Tebbal et al., 2014; Carlucci et al., 2015; Linaldeddu et al., 2015). Among them, two major species are found systematically, notably in France: *Diplodia seriata* (teleomorph *Botryosphaeria obtusa*; Shoemaker, 1964; Lehoczky, 1974; Cristinzio, 1978) and *Neofusicoccum parvum* (Pennycook and Samuels, 1985; teleomorph *Botryosphaeria parva*, Crous et al., 2006), as reported by Larignon et al. (2015). In diseased vines, those fungi have always been isolated from wood and could occasionally be isolated from other tissues such as diseased berries (Steel et al., 2007; Taylor and Wood, 2007; Wunderlich et al., 2011a).

Botryosphaeriaceae species are characterized by a latent phase, when they are present inside the plant as endophytes (Slippers et al., 2007) and by a pathogenic one, when they become virulent and actively colonize the wood (Leal et al., 2024). Due to high enzymatic activity (cell wall-degrading enzymes, especially cellulolytic ones) and toxin production, affected tissues die and local or entire vine drying can occur (Andolfi et al., 2011; Esteves et al., 2014; Claverie et al., 2020; Belair et al., 2023). Given that pathogens are always found in the wood but never in the leaves of infected plants, where typical symptoms occur, it has been also hypothesized that the leaf symptoms may result from extracellular compounds such as exopolysaccharides and proteins (Martos et al., 2008; Bénard-Gellon et al., 2015) produced by fungi in the colonized woody tissues of the trunk. Those compounds, suggested as phytotoxic, would then be translocated to the leaves through the transpiration stream (Mugnai et al., 1999). Besides, some of the Botryosphaeriaceae toxins have been isolated and characterized (Andolfi et al., 2011 for review; Reveglia et al., 2019; Trotel-Aziz et al., 2022).

Infection of vines occurs mainly through pruning wounds, but also at the grafting point in nurseries (Chamberlain et al., 1964; Lehoczky, 1974; Hewitt, 1988; Lehoczky, 1988; Leavitt, 1990), through wounds caused during cultural practices such as disbudding (Molot et al., 2006; Epstein et al., 2008; Makatini, 2014) or from buds (Phillips, 1998; Epstein et al., 2008; Amponsah et al., 2012). However, it was recently shown that artificial infections of annual canes in greenhouse (Reis et al., 2016) and in field (Reis et al., 2019) by Botryosphaeria species lead to the development of necrosis and in some cases, the expression of foliar symptoms. An annual contamination of annual stems by some Botryosphaeriaceae species was hypothesized, resulting from foliar symptoms expression related to vessels obstruction or cambium dysfunction. In our study, our objectives were to better understand the triggering and expression of grapevine severe form of Botryosphaeria dieback in vineyard. Three biological questions were thus asked (1) is the expression of leaf symptoms (leaf drop) conditioned by the presence of pathogens in the annual stems; (2) is there a link between the quantity of blocked vessels (tylosis/gummosis) and the expression of leaf symptoms, and (3) what are the repercussions of such dieback on vine physiology (gene expression and metabolome) and development (establishment and functioning of the cambium and phellogen) at the stem internode level. A transdisciplinary study was thus conducted in a naturally infected vineyard and with different approaches, especially at molecular, cellular and anatomical levels.

## Materials and methods

### Experimental plot

The experiments were performed on grapevine shoots collected in a plot of the EPLEFPA Nîmes-Rodilhan located at Rodilhan (France, GPS coords: latitude 43.829685, longitude 4.450104), in the Costières de Nîmes vineyard. It was planted in 2005 with *Vitis vinifera* cv. Cabernet Franc grafted onto R110 rootstock at a density of 4444 vines/ha, the vines are trained to in bilateral cordon. The soil is clay-limestone type with rolled stones and vines were not irrigated. Climatic data (temperatures and rainfall) collected between 2010 and 2021 are given in **Supplementary File 1**.

### Evaluation of the Botryosphaeria dieback severity in the studied plot

Monitoring and mapping of the expression of the Botryosphaeria dieback have been carried out for each grapevine of the plot since august 2016, and weekly, from flowering to the end of symptom appearance in 2017, 2018 and 2019.

### Sampling for Botryosphaeria isolation by Pasteurian method

In 2018, stems of asymptomatic (control, C) and symptomatic (severe form, D) vines of Botryosphaeriaceae dieback were collected at six different stages: inflorescences visible, flowering, cluster closure, veraison, maturity, leaf fall (**Supplementary Figure 1**). For each stage, new vines (C and D) were sampled. Thirty stems of 30 to 40 cm length were collected per stage, randomly from the plot, and 40 fragments per stem were cut from the three first basal internodes to be analyzed. In this approach, 20 fragments became from internodes 1 and 2 (in mix) and 20 others became from the 3^rd^ internode.

### Sampling for cytological and molecular analysis

In 2018, stems of asymptomatic (control, C) and symptomatic (severe form, D) vines of Botryosphaeriaceae dieback were collected at three different stages: flowering (T1: pre-symptomatic stage), cluster closure (T2: early expression of symptoms) and late veraison (T3: severe symptomatic stage with leaf drop; **Supplementary Figure 1**). At flowering stage, no symptoms were observed leading to sample D stems on vines expressing disease in 2017. For each stage, 5 new vines (C and D) were sampled. For each of the five vines selected for both modalities C and D, two stems were sampled: one for the anatomical characterization (type A) and the second (type B) for molecular analyses (**Supplementary Figure 1**).

#### Sampling for macroscopic and microscopic analyzes

Stems of type A, without inflorescences, clusters and leaves were stored at 4°C. Then, samples were taken from the first three basal internodes for macroscopic and microscopic observations of vascular tissues.

#### Sampling for gene and metabolomic analyzes

Pieces of internodes 1, 2 and 3 (number 1 being closest to the base) were sectioned from stems of type B. The xylem area was detached from the phloem/bark (thereafter called “Xylem” or “X” and “Phloem”, “P” or “bark”, respectively) part, individually snap-frozen and then stored at -80°C. As small quantities of tissues were sampled by internode, we mixed tissues from the same internode of the five sampled vines. Each sample was ground in liquid nitrogen using a ball mill (Retsch MM400) and divided in two sub-samples: one for molecular analyzes (PCR and qRT-PCR), and one for metabolic analysis (GC-MS).

### Detection of Botryosphaeria pathogens

#### Pasteurian analysis: pathogen isolation and colony discrimination

Stems of three first internodes from the five asymptomatic and symptomatic plants were sampled at 6 stages. Four pieces of approximately 3x1x1 mm in size from each internode were placed in Petri dishes containing malt agar medium (15 gL^-1^ cristomalt, 20 gL^-1^ agar, 200 mgL^-1^ of chloramphenicol) and incubated in the dark at room temperature (Larignon and Dubos, 1997). Xylem and bark were weekly observed and pictures were acquired (Canon Power Shot G7) for 4 weeks for visual examination. The color, the hyphal and pycnidiospore morphology, and the aerial mycelium of the colony were examined under light microscope (Larignon et al., 2001).

#### Molecular identification: DNA extraction and PCR tests

The genomic DNA was extracted from molecular analyses sample powder of X and P tissues, by following the extraction protocol of Mundy et al. (2018). The genomic DNA of X and P samples was extracted using the DNeasy Plant Mini kit (Qiagen, Hilden, Germany). The quality of DNA was checked by agarose gel electrophoresis and the quantity was determined by measuring the absorbance at 260 nm.

Amplicons were generated by PCR using the Phusion High-Fidelity DNA polymerase, 20 ng of DNA, and the primers ITS1-F and ITS4, as described by White et al. (1990) and Udayanga et al. (2012) (**Supplementary Table 1**). A nested PCR was performed taking 5 μL of the 1:100 diluted PCR1 template and using primers BOT100F and BOT472R, as described by Ridgway et al. (2011) (**Supplementary Table 1**).

### Macroscopic observation and image analysis obstruction of xylem vessels

#### Macroscopic observation and image acquisition

From the collected internodes 1, 2 and 3, cross sections were performed and observed under a macroscope (ZEISS, AXIOZoom.V16). Two images were acquired using the “SNAP” command (ZEN2.3 system software); one with samples exposed to white light (RGB experiment settings), and one with samples exposed to blue light only (DAPI experiment settings, excitation 365 nm, emission 445-450 nm).

#### Image analysis to estimate the percentage of xylem vessels obstructed

A semi-automatic analysis was performed to establish the proportion of obstructed vessels among the total number of vessels. First, the total number of vessels (both obstructed or not obstructed) was counted automatically with an ImageJ macro. The image acquired with DAPI settings was processed with ImageJ 1.52 software with the Fiji package. Images were contrasted manually, then an automatic threshold was created (Auto Local Threshold parameter: method Phansalkar), thus converting the picture in either black or white pixels, without grey shades. Then using the “Analyze particles” setting, the total number of vessels was assessed (parameters: size: 35-1000; circularity: 0.35-1.00). The number of obstructed vessels was assessed manually on the RGB image using the multi-point count tool from the Fiji package.

The ratio of obstruction was calculated as follows:


(Number of obstructed vessels/Total number of vessels)*100

### RNA extraction and real-time qRT-PCR analysis

#### RNA extraction

Total RNA was isolated from 3 x 100 mg of X and P powder using the PureLink Plant RNA Purification Reagent (Invitrogen, Cergy Pontoise, France). The manufacter’s protocol was followed until the phase of separation with the chloroform: isoamylic alcohol (24:1). Then, one volume of ethanol 70% was added to the aqueous solution, before purification using the NucleoSpin RNA kit (Macherey-Nagel, Düren, Germany). The quality of RNA was checked by agarose gel electrophoresis and the quantity was determined by measuring the absorbance at 260 nm for each sample and adjusted to 100 ng µL-1. First-strand cDNA was synthetized from 150 ng of total RNA using the Verso cDNA Synthesis kit (Thermo Fisher Scientific, Inc., Waltham, MA, United States).

#### Real-time qRT-PCR

Real-time polymerase reaction (PCR) was performed using Absolute Blue qPCR SYBR Green (Thermo Fisher Scientific, Waltham, MA, USA), in a CFX96 real-time PCR detection system (Bio-Rad, Hercules, CA, USA). The thermal profile was 10 s at 95°C (denaturation) and 45 s at 60°C (annealing/extension) for 40 cycles. The specificity of PCR amplification was checked using a heat dissociation curve from 65 to 95°C following the final cycle. For each experiment, PCR reactions were performed in duplicate. Expression of two reference genes (*EF1-α* and *60SRP*) and of genes encoding enzymes involved in the sucrose metabolism and signalization (*αAMY*, *βAMY*, *CWINV* and *SUC27*), the plant defense responses (*Cal-S7*, *GLU*, *GST5*, *PAL*, *POX4*, *PR6*, *STS*, *TL* and *WAT1*), detoxification and stress tolerance (*epoxH2* and *SOD*), vascular cambium (*CDKB2*, *ERF5*, *MOL* and *WOX4*) and cork cambium (*APL*, *EBP1*, *HA3*, *SHR*, *PSKR1* and *PSKR2*) was tracked by quantitative reverse transcription-PCR (qRT-PCR) using the primers listed in **Supplementary Table 2**.

### Metabolic analysis (GC-MS)

GC-MS analyses were performed from 50 mg of X and P powder (Moret et al., 2019). Briefly, samples were extracted for 10 min at 4°C with shaking in 1 mL of water:acetonitrile:isopropanol (2:3:3) and ribitol 4 µg/mL. After centrifugation (20,000 g, 5 min), 100 µl supernatant were collected and dried for 5 h in a SpeedVac vacuum centrifuge. Samples were derivatized as described in Krzyżaniak et al. (2018) and analyzed using an Agilent 7890A gas chromatograph coupled to an Agilent 5975C mass spectrometer. A Quality Control (QC) made of all samples merged together was injected 3 times and was used as a standard to evaluate the quality of the analysis.

### Microscopic observation of bark and xylem tissues

Segments (2 by 5 mm) were randomly excised from the same C and D stems used for macroscopic analyses.

#### After inclusion in paraffin and aniline blue staining

The samples were fixed into a fresh mixture of 4% paraformaldehyde in potassium phosphate buffer (PBS 10mM, pH 7.4, with addition of tween 20 1‰ only in the first 1 h bath) and then embedded in paraffin (Colas et al., 2010). Wax sections at 10 µm thickness were obtained using a microtome and deposited on silanized slides. The samples were then dewaxed by 2 Q Path^®^ Safesolv baths (20 min each at room temperature), gently rinsed with a pipette in ethanol 90° (1 min at room temperature) and then gradually rehydrated; deposits of drops of ethanol 70°, 50° and 25° were gradually made and each was left for 1 min in contact with the sections. Finally, ultra-pure water was put in contact on the slide during 2 min in order to completely rehydrate the sample.

Aniline blue staining (1% in 3% acetic acid) was carried out by leaving the drop of dye 30 s on the samples, then rinsing once 10 s with 3% acetic acid and then twice 10 s with ultra-pure water in order to remove excess dye. Finally, the samples were observed under a bright-field light microscope (Leitz DM RB, Leica), and images were acquired using a camera (Nikon Digital Sight), with respect of the same shooting parameters for each observed sample.

#### After inclusion in Epon resin and toluidine blue staining

The samples were fixed immediately in 2.5% glutaraldehyde (in 0.1 M sodium- phosphate buffer, pH 7.2, 1% sucrose, 1 ‰ Tween 20) for 20 to 25 min under vacuum, then overnight at 4°C with gentle rotation (without Tween 20). Samples were washed twice (10 min) in the same buffer and postfixed in 1% osmium tetroxide (OsO_4_) in the same buffer for 1 h at 4°C. Then, samples were washed in phosphate buffer, dehydrated in a graded ethanol series, and treated with propylene oxide. Dehydrated samples were subsequently embedded in Epon (Merck, Darmstadt, Germany) and sectioned using a Reichert Ultracut E microtome (Leica, Reuil-Malmaison, France) with glass or diamond knives (Diatome, Bienne, Switzerland). Semithin (0.5 μm) sections from the tissue blocks were stained with 1% aqueous toluidine blue (in 1% sodium tetraborate) and examined under a bright-field light microscope (Leitz DM RB, Leica) in order to observe anatomical characteristics of both bark and xylem.

### Data processing and statistical analysis

#### Anatomical studies

Data analysis were carried out using Past 4.08 software. A Chi-square (X²) test of homogeneity was used to compare the distribution of obstructed to non-obstructed vessels in shoot of asymptomatic and symptomatic stems. Moreover, a Kruskal Wallis test (p<0.05) was used to analyze and compare the proportions of obstructed vessels between internodes and sampling period.

#### Expression of genes

For each gene and for each modality, a mean Cq value was obtained. Relative gene expression (RE) was determined with the 2-ΔΔCq method using CFX Manager 3.0 software. For every sample, ΔΔCq was the ΔCq difference between 2 samples (diseased *vs* control). These values were used to generate a gene expression heat map through Past 4.08 software. Hierarchical clustering (ward’s method, Euclidian distance) was applied to group samples with similar expression level.

#### Metabolomic studies

GC-MS data were processed as described in Moret et al. (2020). Briefly, Data files in NetCDF format were analyzed with AMDIS software. A home retention indices/mass spectra library and standard compounds were used for metabolite identification. Peak areas were determined with the Targetlynx software (Waters). AMDIS, Target Lynx in splitless and split 30 mode data were compiled into a single Excel file for comparison and peak areas were normalized to ribitol and fresh weight. GC-MS data were then pre-processed and filtered RSD (relative standard deviation) on QC samples was calculated for each compound. Only features with RSD > 30% and non-aberrant QC were conserved for further analysis. Missing values were replaced by half the lowest value, corresponding to limit detection of the method. PCA (Principal Component Analysis) and PLS (Partial Least Square) analyses were made on the processed data. Mean, fold change and two-sided Student’s T-test p-value (p<0.05) were calculated and compiled into a data frame that was used for the Volcano plots (Goodacre et al., 2007; Vinaixa et al., 2012; Worley and Powers, 2013; Schiffman et al., 2019). Significant compounds highlighted by Volcano plots (T-test p<0.05) were verified and compounds with aberrant concentrations across samples were removed.

## Results

### Health status of vines

The results of the monitoring of diseased vines within the plot are displayed in **Table 1**. During the 4 years of monitoring, the dead/absent/complant vines number increased significantly from 4% to 18%. From 2017 (7.65%) to 2018 (10.84%) it was 1.4-fold that is lower compared to other years (2016-2017: 1.87-fold; 2018-2019: 1.72-fold). Eutypa dieback occurrence increased from 1.17 to 2.63% from 2017 to 2019. Apoplexy occurrence was low, of about 0.5% from 2016 to 2019. Botryosphaeria dieback occurrence increased from 6.37% in 2016 to 14.36% in 2017, and then decreases from 9.33% in 2018 to 4.64% in 2019. Therefore, the foliar expression in 2018 was considered as moderate when comparing with 2016, 2017 and 2019.

**Table 1:** Sanitary situation of the plot during the different vintages.

% vines	2016	2017	2018	2019
Dead/absent/complant	4,08	7,65	10,84	18,27
Eutypa dieback	nd	1,17	1,79	2,63
Apoplexy	0,61	0,39	0,61	0,39
Botryosphaeria dieback	6,37	14,36	9,33	4,64
Unproductive vines	11,06	23,58	22,57	25,92

### Botryosphaeriaceae fungi could be detected in the three basal internodes in both bark (phloem) and xylem tissues

#### Botryosphaeriaceae fungi are isolated both in the bark and the xylem

**Table 2** summarizes the analysis of 1200 fragments of either bark or xylem. In 2018, Botryosphaeriaceae were isolated whatever the sampling time, with a maximum detection at maturity in both phloem (bark) and xylem. Botryosphaeriaceae isolates were more abundant from bark samples than from xylem samples (i.e., 14 times more at the “inflorescences visible” stage). The number of isolates was similar in both tissues at flowering stage, but two times more abundant in the bark than in the xylem at maturity stage.

**Table 2:** Percentage of vine fragments in which Botryosphaeria were isolated in 2018 from the bark and xylem tissues” of stems.

	Bark (phloem)	Xylem
Inflorescences visible	1.17	0.08
Flowering	0.41	0.5
Cluster closure	1	1.25
Veraison	5.41	0.67
Harvest Maturity	6.42	2.58
Leaf fall	4.92	1.33

#### Botryosphaeriaceae fungi are detected by PCR in phloem and xylem tissues of internodes of control and diseased vines

Botryosphaeriaceae were present in both control (C) and diseased (D) vines, in both phloem (P) and xylem (X), whatever the internode (1, 2 and 3) and the sampling period (**Table 3**). The detection of Botryosphaeriaceae is less frequent at T3 (late veraison stage; 6 out of 12 samples) than at T1 (flowering; 10 out of 12 samples) and T2 (cluster closure; 11 samples out of 12).

**Table 3:** PCR detection of fungi and Botryosphaeriaceae (“Botryo”) according to internode and tissue (P: phloem; X: xylem) levels and sampling times (T1, from pre-symptomatic stems; T2 and T3 from symptomatic ones).

			T1		T2		T3	
	tissue	internode	Fungi	Botryo	Fungi	Botryo	Fungi	Botryo
**C**	P	1	✔	✔	✔	✔	✔	✔
		2	✔	✔	✔	✔	✔	✔
		3	✔	✔	✔	✔	✔	✔
	X	1	✔	✔	✔	✔	✔	✔
		2	✔	✔	✔	✔	✔	–
		3	✔	✔	✔	✔	✔	–
**D**	P	1	✔	–	✔	✔	✔	–
		2	✔	✔	✔	✔	✔	–
		3	✔	✔	✔	✔	✔	–
	X	1	✔	✔	✔	✔	✔	✔
		2	✔	✔	✔	✔	✔	–
		3	✔	–	✔	–	✔	✔

Minus indicates that fungi were not detected, whereas the symbol “✔” indicates that a PCR amplicon was obtained at the expected size.

### The quantity of blocked vessels and the foliar disease expression are not related to the grouped or localized repartition of the obstructed vessels

**Figure 1:**
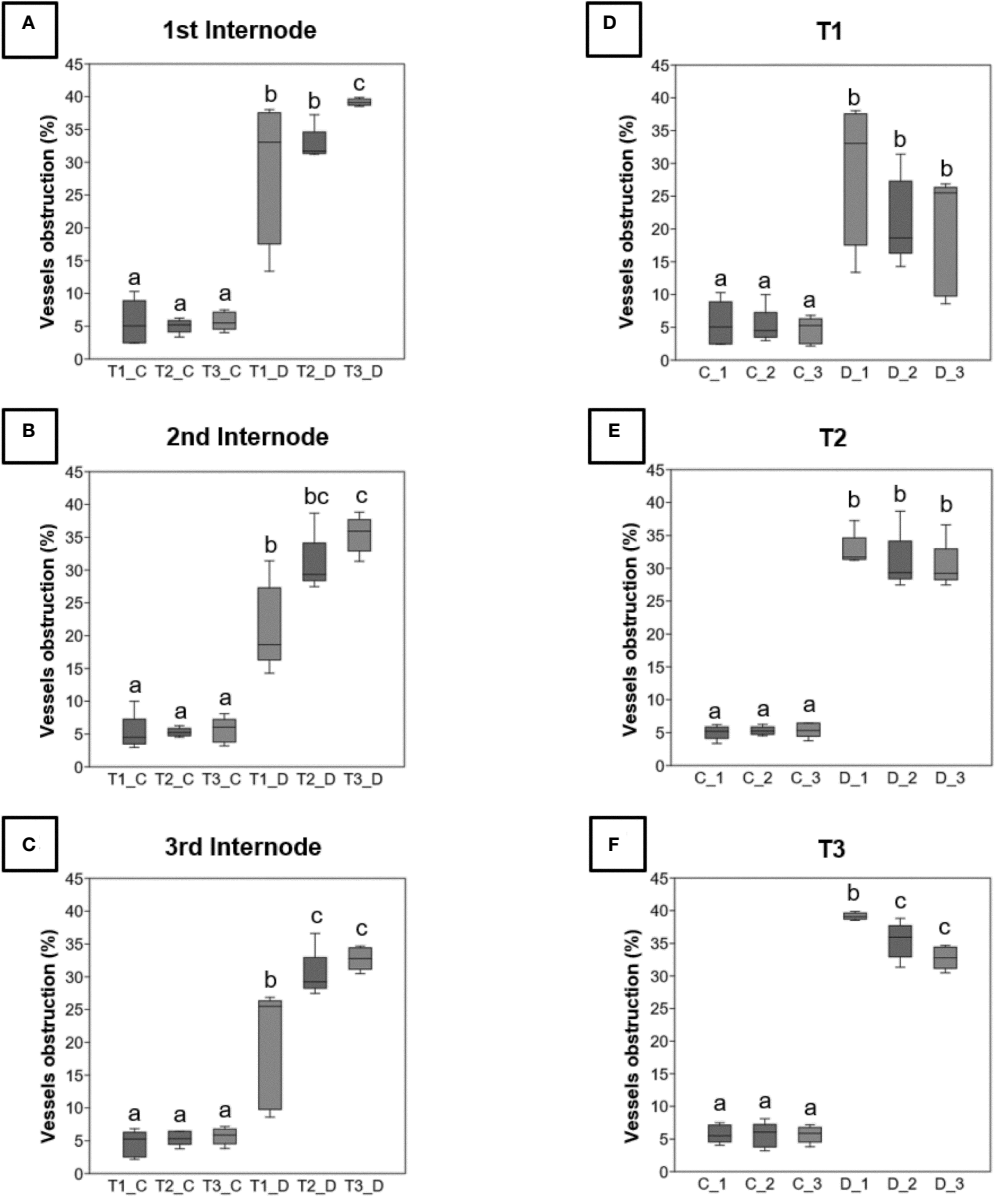
Rate of obstruction of xylem vessels detected by analysis of images acquired from sections of 3 internodes (_1, _2 and _3) taken from control **(C)** or diseased **(D)** stems for the three times of sampling (T1, T2 and T3). These rates are expressed as a percentage and were evaluated on the three levels of internodes **(A-C)**, and at the three sampling times **(D-F)**. Differences between control and diseased for each time were statistically significant (p<10^-6^, chi square test) and differences between internodes were noted in figure by letters (p<0.05, Kruskal-Wallis test).

In diseased vines, a higher obstruction ratio was observed in the three internodes (**Figures 1A–C**) for the three sampling periods (**Figures 1D–F**) in comparison to control plants. For a same internode, the obstruction percentages were significantly higher at the late symptomatic stage (**Figures 1A–C**, T3_D samples) than at the pre-symptomatic one (**Figures 1A–C**, T1_D samples) for diseased vines only. For each sampling time, there was no significant difference among internodes (**Figures 1D,E**), except for the internodes 2 and 3 that were significantly less obstructed than the internode 1 at the late symptomatic stage (**Figure 1F**, D_2 and D_3 *vs* D_1 samples).

**Figure 2:**
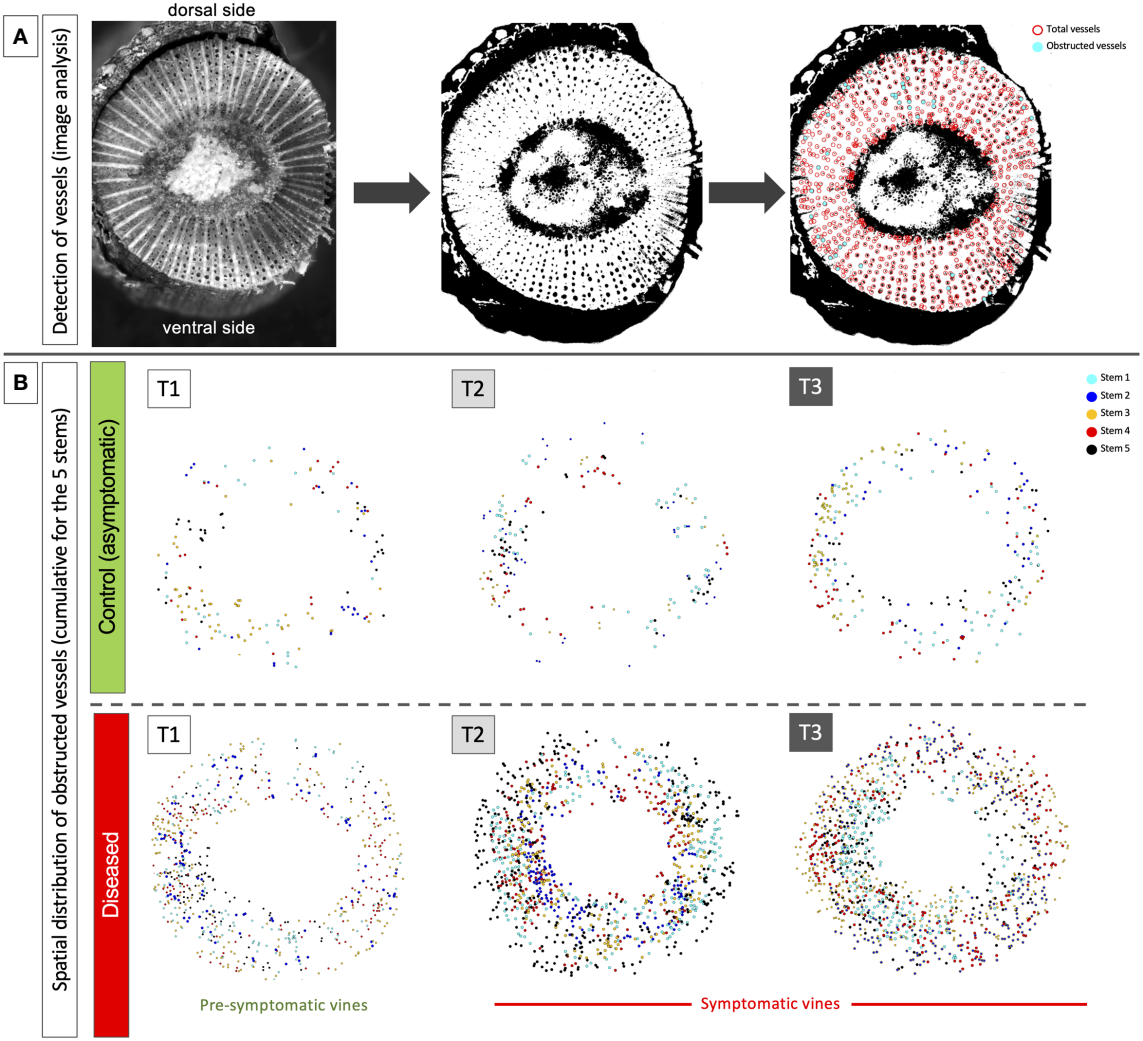
Example of results acquired by image analysis in the second internode of the stems. **(A)** illustration of the process carried out from image acquisition, contrast/thresholding and semi-automatic detection of total (in red) and obstructed vessels (in cyan). **(B)** illustration of the temporal and spatial cumulative distribution (stems 1 to 5) of obstructed vessels at the circumference of the stems and along the dorsal/ventral and lateral axes. The results were presented for the three sampling times: T1 (pre-symptomatic), T2 (appearance of the first symptoms) and T3 (leaf drop). Each layer obtained for each stem taken in a same modality **(C** or **D)** presents a different color: cyan for stem 1, blue for stem 2, orange for stem 3, red for stem 4 and black for stem 5.

The image analysis of the spatial distribution of obstructed vessels in the circumference of the internode revealed a random distribution in each cane whatever the stage (pre-symptomatic or symptomatic stage; **Figure 2** and **Supplementary Figure 2**).

### Botryosphaeria dieback impacts grapevine stems before the onset of leaf symptoms

#### Modification of gene expression is enhanced by the appearance of foliar symptoms

**Figure 3:**
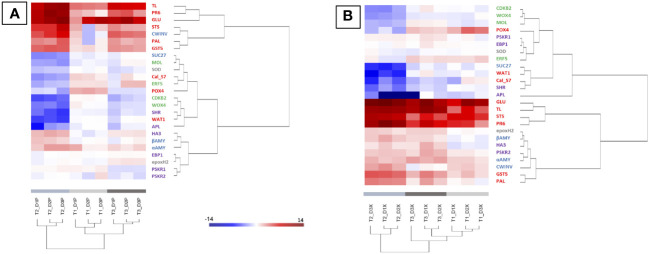
Heatmap generated from qPCR gene expression analysis in diseased vine stems compared to control one. Expression of 25 genes related to plant defense (in red), detoxification and stress tolerance (in grey), sucrose metabolism and signalization (in blue), or cambial (in green) and phellogen (in purple) activity was assessed in phloem **(A)** and xylem **(B)** tissues. Hierarchical clustering (ward’s method, Euclidean distance) was applied to group samples with similar expression levels, emphasizing the overall changed expression during pre-symptomatic (T1, light grey) and symptomatic (T2, medium grey and T3, dark grey) stages. The color intensity indicates expression levels; red: up-regulation, blue: down-regulation.

In xylem (X) and phloem (P), targeted genes on sucrose metabolism and signalization, plant defense response and detoxification process, and secondary meristems were studied on the 3 internodes before and during the expression of foliar symptoms. A hierarchical clustering (heatmap) was made in order to group samples, in P or X tissues, with similar expression levels (**Figure 3**). Regardless of the tissue observed (P or X), the samples from times T1, T2 and T3 were clearly separated. Overall and regardless of the plant tissue, defense genes such as *TL*, *PR6*, *GLU* and *STS* were among the most up-regulated, followed by genes related to detoxification (*GST5*) and to the degradation of storage or transit sugars (*αAMY* and *CWINV*).

Even if the most regulations (up and down) were observed for the time T2 (start of appearance of symptoms), it is interestingly to note that regulations (up and down) were also detected before the expression of leaf symptoms (T1; **Figure 3**, light grey). At this stage, genes related to carbohydrate metabolism (*aAMY* and *CWINV*) and defense responses (*GLU, PR6, GTS5, POX4, STS* and *TL*) were up-regulated in both X and P tissues. Moreover, the *Cal-S7* gene was up-regulated in P tissues (**Figure 3A**) and down-regulated in X tissues (**Figure 3B**). At T1, no gene modifications related to the formation of the secondary meristem were detected except for *APL*, repressed in X tissues. At the early symptomatic stage (emergence of symptoms at T2), the expression of the majority of targeted genes was modified in X and P tissues of D vines (**Figure 3**). A down-regulation of genes associated to the cambium (*WOX4, CDKB2* and *MOL*), the phellogen (*APL* and *SHR*) and sucrose signalization (*SUC27*), and an up-regulation of genes associated to carbohydrates (*aAMY, βAMY* and *CWINV*), plant defense (*GLU, PR6, GTS5, STS, PAL* and *TL*) and phellogen (*HA3*, and *PSKR2*) were observed (**Figure 3**, medium grey). Changes at the late symptomatic stage (T3) were similar to changes at T2, except for genes involved in the formation of secondary meristems and showing less changes (**Figure 3**, dark grey).

#### Changes of the grapevine metabolome is tissue- and time-specific with Botryosphaeria dieback

**Figure 4:**
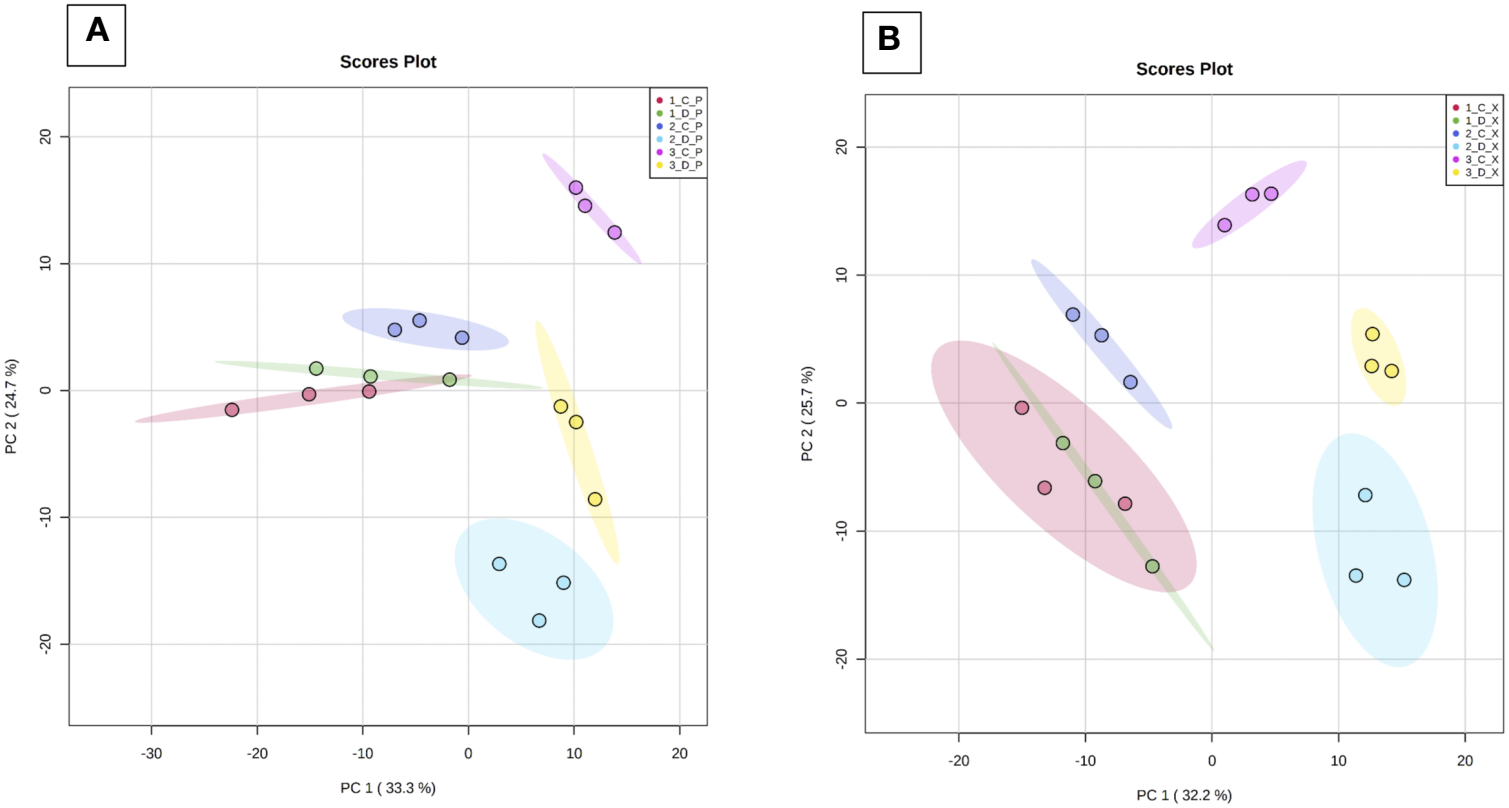
Principal component analysis (PCA) of GC-MS data. Analyses were performed from phloem (Panel **A**) and xylem (Panel **B**) extracts of the three basal internodes of control and diseased stems. Samples were collected at T1 (before symptom expression), T2 (onset of symptom expression) and T3 (full symptom expression). 1 to 3: sampling time (T1, T2, T3), C/D (control/diseased), P: phloem and X: xylem. PC1: Time of sample collection; PC2: health state.

Hierarchical Cluster Analysis (HCA) analysis performed on the whole sample set allows to clearly separate the phloem (P) and xylem (X) samples in two clusters (**Supplementary Figure 3**). PCA analyses were therefore performed on GC-MS data of each tissue (**Figure 4**). Samples were separated depending on time (PC1, 32.2%), and to a lesser extent depending on health state (PC2, 25.7%). For both tissues, at time T1 (before symptom expression), there is no separation of C and D samples (red and green ellipses, respectively), suggesting a similar metabolic profile. At T2 and T3, all groups are different and different from T1, indicating that their metabolic profile evolves over time with disease expression. For both tissues, variability is low whatever the modality and the sampling time.

**Figure 5:**
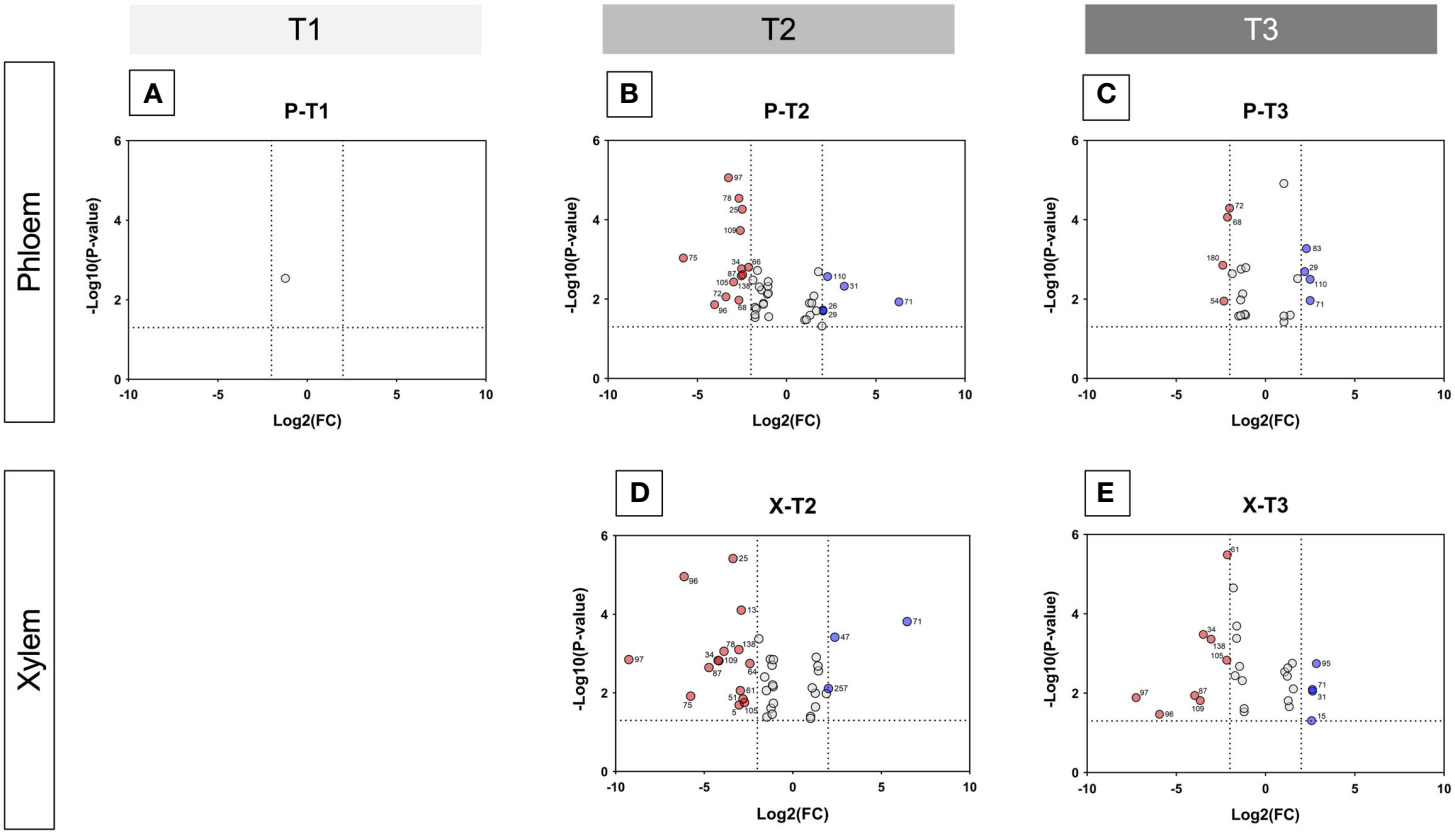
Volcano plots showing metabolite variations in internodes. For each metabolite, fold change (C/D) and Student’s T test p-value were calculated between C and D samples (C control, D diseased) for both xylem and phloem tissues of the three internodes of the base of stems, and for T1 (before symptom expression), T2 (onset of symptom expression) and T3 (full symptom expression) sampling times. The results are displayed on a Volcano plot, with p< 0,05 and FC > 2 thresholds. Each metabolite is displayed by a point and corresponds to (i) p-value< 0.05 and FC > 2 (in red and blue), (ii) p-value< 0.05 and FC< 2 (in grey). Metabolites that were significant and validated the fold change threshold are annotated on the plot with their ID number. In red: metabolites in higher concentration in D than in C tissues; in blue: metabolites in higher concentration in C than in D tissues. **(A, B)**: Volcano plots for xylem samples collected at T2 (onset of symptom expression) and T3 (full symptom expression), respectively. **(C-E)** Volcano plots for phloem samples collected at T1 (before symptom expression), T2 (onset of symptom expression) and T3 (full symptom expression), respectively.

**Figure 6:**
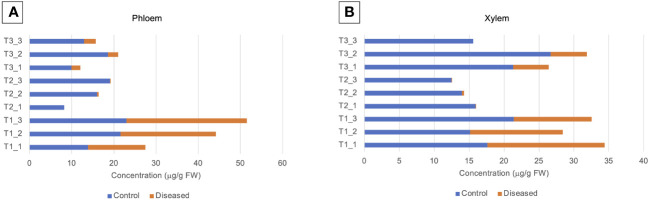
Level of 1-kestose (μg/g FW) in **(A)** phloem and **(B)** xylem samples.1-kestose was analyzed by GC-MS. Phloem (P) and xylem (X) tissues were obtained from samples collected before symptom expression (“1”), at the onset of symptom expression (“2”) and at full symptom expression (“3”). Samples were obtained from control (C) and diseased (D) internodes 1, 2, 3 located at the base of stems.

Volcano plants represent the distribution of compounds for which the concentration is significantly different in X and P tissues between C and D samples (**Figure 5**, **Supplementary Tables 3**, **4**). The number of compounds differentially accumulated in P and X tissues is of 1 and 0 for P and X, respectively at T1, of 42 and 39 for P and X, respectively, at T2, and of 22 and 27 for P and X, respectively, at T3. At T1, glycosylsalicylate was less than two-times more accumulated in P tissues of D plants than in P tissues of C plants. At T2, there was two-times more compounds accumulated in samples from D plants than in samples from C plants. In P tissues, 1-kestose, digalactosylglycerol and threonate were more accumulated in tissues from C plants than from D plants, and mannitol, *trans*- and *cis*-resveratrol and leucine were more accumulated in tissues from D plants than from C plants (**Supplementary Table 3**). In X tissues, 1-kestose and galactinol were more accumulated in tissues from C plants than from D plants, and *trans*- and *cis*-resveratrol, mannitol and piceid were more accumulated in tissues from D plants than from C plants (**Supplementary Table 4**). At T3, there was more compounds accumulated in tissues from D plants than from C plants. In P tissues, 1-kestose, threonate and phenylalanine were more accumulated in tissues from C plants than from D plants, and glutamine and isoleucine were more accumulated in tissues from D plants than from C plants (**Supplementary Table 3**). In X tissues, raffinose, digalactosylglycerol and 1-kestose were more accumulated in tissues from C plants than from D plants, and *trans*- and *cis*-resveratrol, and piceid were more accumulated in tissues from D plants than from C plants (**Supplementary Table 4**). The concentration of 1-kestose was determined in all samples as significantly accumulated in tissues from C samples (**Figure 6**). At T1, the concentration of 1-kestose was similar in X and P tissues from C and D samples (C/D ratio from 0.8 to 1, and from 1 and 1.9 in P and X tissues, respectively). At T2, the concentration sharply decreased in X and P tissues from D plants (not detected in internode 1 of P). At T3, the concentration increased (except in X of internode 3: not detected) in X and P tissues, without reaching T1 values (C/D ratio from 4.6 to 7.5, and from 4.1 to 5.1 in P and X tissues, respectively).

### Botryosphaeria dieback strongly affects the anatomy of bark and xylem tissues

A similar pattern was observed in all internodes from D samples, revealing deep tissue disorganization from pre-symptomatic vines to the full expression phase.

#### Annual stems present deep alterations in phloem, xylem and secondary meristems establishment and/or functioning

**Figure 7:**
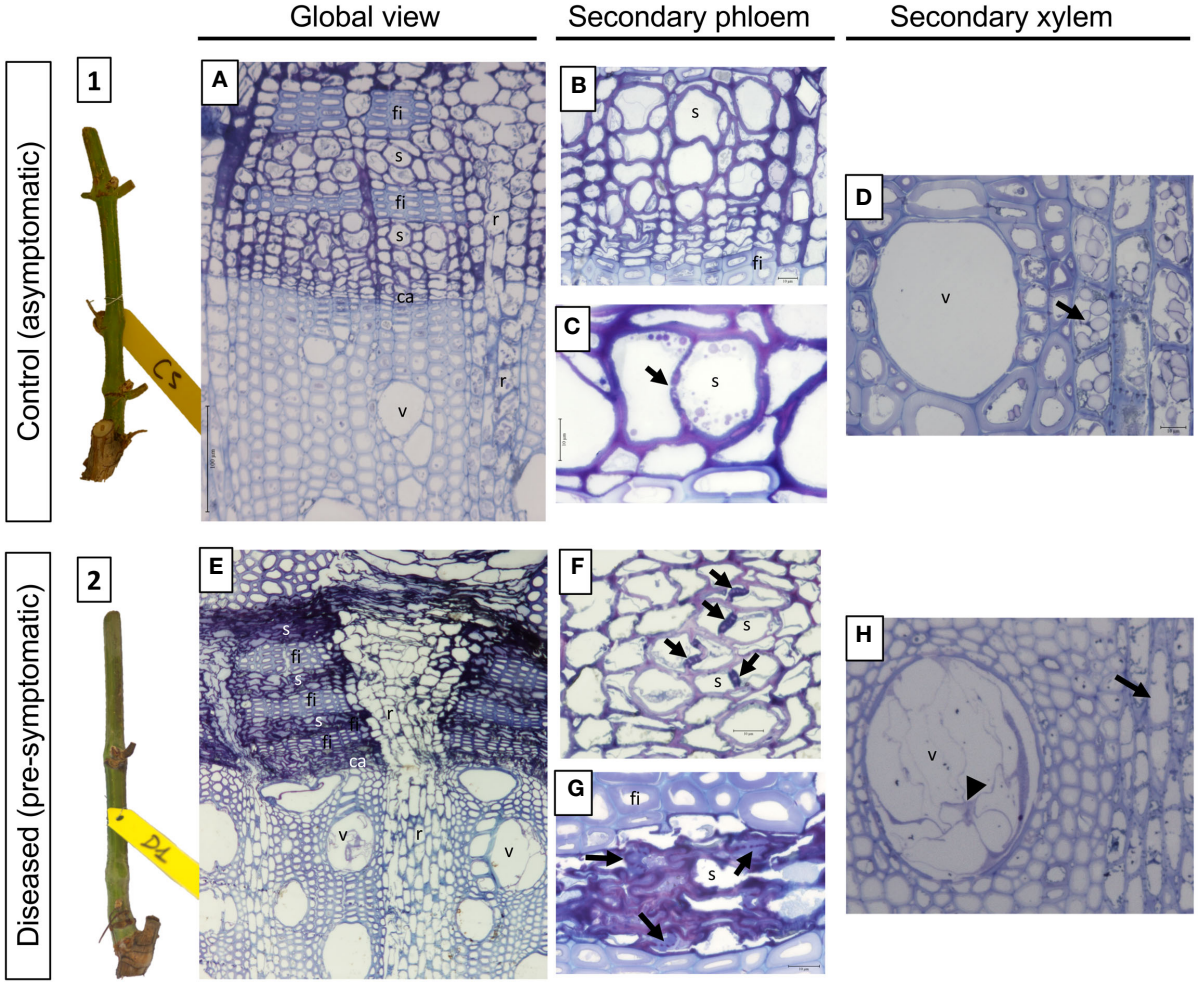
Phenotypic and structural observations of grapevine stems harvested at T1 (pre-symptomatic stage) from control (1, **A-D**) or diseased (2, **E-H**) plants. A representative picture of the phenotype of the stems sampled is given in illustrations 1 (control) and 2 (pre-symptomatic). The observation of semi-thin sections stained with toluidine blue also allows the comparison of the anatomical structure of the stems. Thus, a global view is given in A (control) and B (diseased), while focuses are made on the secondary vascular tissues that are the phloem (B and C: control; F and G: diseased) and the xylem (D: control; H: diseased), respectively. The photographs produced are representative of a minimum of 15 blocks observed per internode level (5 stems with 3 blocks per cane and per internode). ca, vascular cambium; fi, phloem fibers; r, vascular rays; s, sieve plate; v, xylem vessel.

At T1, the herbaceous stems were collected before the onset of leaf symptom expression (**Figure 7**).

Regarding its anatomy, the overall view of the C sample (**Figure 7A**) has a characteristic structure of the Euvitis section of Vitis genus (Metcalfe and Chalk, 1950; Esau, 1965). The secondary phloem appears in blocks separated from another one by wide rays (**Figure 7A**, r). Within the blocks, tangential strata of sieve elements (**Figure 7A**, s) with associated companion cells alternate with tangential bands of fibers (**Figure 7A**, fi). Separating the secondary P from the secondary X, the presence of the vascular cambium is clearly observed (**Figure 7A**, ca). At the level of the secondary P (**Figure 7B**), the sieve tubes and their companion cells are distinguished, indicating that this tissue is functional in the transport of the elaborate sap. Moreover, functional sieve plates can also be observed (**Figure 7C**, arrow). The secondary X presents functional, unobstructed vessels (**Figures 7A, D**), and many amyloplasts could be observed in parenchyma rays (**Figure 7D**, arrow).

In the stems sampled on declining vines (pre-symptomatic), clear anatomical alterations were observed in both secondary P and X (**Figure 7E**). In the secondary P, even if the layers of fibers appear conform to what is observed in C samples, vascular cells are disorganized. Indeed, in some areas the sieve plates appeared blocked by a material more or less intensely stained with toluidine blue (**Figures 7F, G**, arrows). In the meantime, the P vascular cells appeared collapsed in some layers, crushed and therefore non-functional (**Figure 7G**). Finally, in the secondary X some vessels were obstructed by the presence of tylosis (**Figures 7E, H**). It clearly also appeared that, unlike C samples, the parenchyma rays were empty of any amyloplast (**Figure 7H**, arrow).

**Figure 8:**
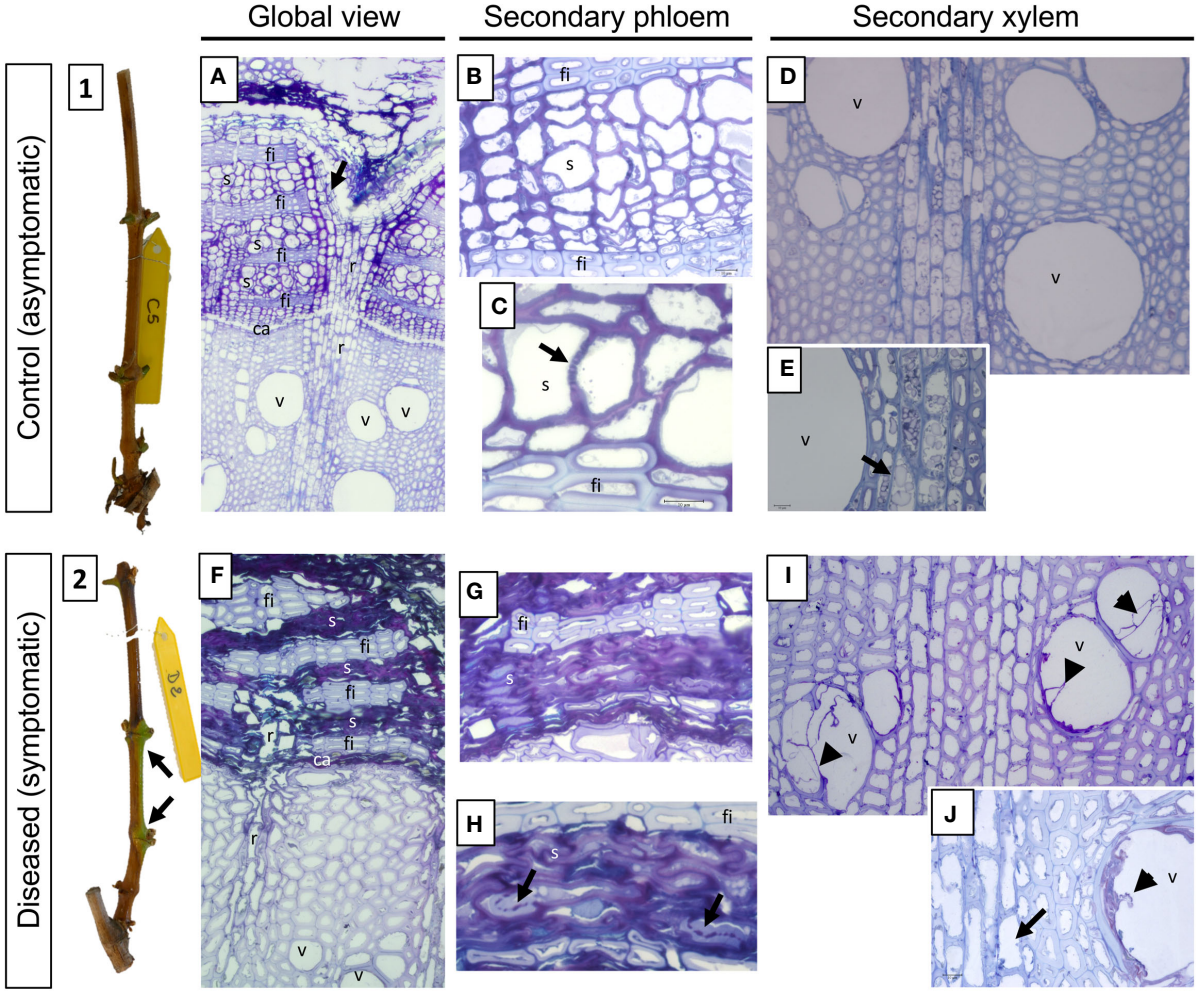
Phenotypic and structural observations of grapevine stems harvested, at T2 (symptomatic stage), from control (1, **A-E**) or diseased (2, **F-J**) plants. A representative picture of the phenotype of the stems sampled is given in illustrations 1 (control) and 2 (leaf drop). The observation of semi-thin sections stained with toluidine blue also allows the comparison of the anatomical structure of the stems. Thus, a global view is given in **A** (control) and **B** (diseased), while focuses are made on the secondary vascular tissues that are the phloem (**B**, **C**: control; **G**, **H**: diseased) and the xylem (**D**, **E**: control; **I**, **J**: diseased), respectively. The photographs produced are representative of a minimum of 15 blocks observed per internode level (5 stems with 3 blocks per cane and per internode). ca: vascular cambium; fi: phloem fibers; r: vascular rays; s: sieve plate; v: xylem vessel.

At T2 and in normal development (C plants, **Figure 8.1**), the stem will transform from an immature state with a green cortex (herbaceous stage), to a stem with collapsed and brownish epidermis created by the developing periderm from an active phellogen (the cork cambium, a lateral meristem that creates the periderm). Indeed, on the 5 C stems taken, all had a periderm (data not shown). Structural observations of C stem (**Figure 8A**) revealed that the initial periderm is formed, normally, within the secondary phloem (**Figure 8A**). Periderm is the secondary protective tissue that replaced the epidermis. It consisted of phellem (cork), phellogen (cork cambium) and only few layers of phelloderm (Esau, 1948). Under the periderm, we observed the same tissues as those described previously with the secondary phloem, separated from the secondary X by the vascular cambium (**Figure 8A**, ca). As for the previous sampling time, the secondary phloem appears functional (**Figure 8B**), with the presence of sieve tubes and unobstructed sieve plates separating neighboring vascular elements (**Figure 8C**, arrow). In addition, the xylem also appears to be functional (**Figure 8D**), with the presence of empty, unobstructed vessels and a large number of amyloplasts in the parenchymal rays (**Figure 8E**, arrow), suggesting an efficient starch storage.

In D vines, periderm development was complete for two canes, incomplete for one and not differentiated for two (data not shown). As example, green islands found on the stem of a D cane (**Figure 8.2**) consist on an incomplete periderm formation and subsequent uneven cane maturation. At the structural level, we therefore observed a defect in the placement of the phellogen, reflected by the absence of this meristem at the level of the outermost secondary phloem layers (**Figure 8F**). Moreover, as at the previous sampling time, and whatever the inter-node, the secondary phloem appears non-functional from a vascular point of view (**Figures 8F–H**). Indeed, the vascular cells are totally collapsed and it even becomes almost impossible to distinguish them individually (**Figures 8G, H**). Occasionally, the presence of sieve plates is still observed which appears obstructed by a component weakly stained with toluidine blue (**Figure 8H**, arrows). In the secondary xylem we also observed the presence of vessels obstructed by tylosis (**Figures 8I, J**) and the lack of amyloplast in the parenchymal rays (**Figure 8J**, arrow), indicating a defective storage in D vines. Finally, in the internodes at the base of the canes (internode 1), we were able to demonstrate the presence of fungal hyphae in the secondary phloem (**Supplementary Figures 4C-E**, arrows). In all cases, the presence of these hyphae was found in the tissue layers closest to the vascular cambium and not in the most peripheral or internal layers.

#### Callose deposition on sieve plates affects the sap conduction and phloem functioning in diseased pre-symptomatic and symptomatic vines

The potential alterations in the secondary P were observed and identified after aniline blue staining from samples of C and D plants included in paraffin. In samples from C plants, the sieve tubes appear functional (**Supplementary Figures 5A, B**), the sieve plates are visible (**Supplementary Figure 5B**, arrows) and a simple pale blue staining (ie. background staining) is detected. At T1, in samples of D plants, an intense blue staining is observed at the level of the cell walls of the sieve tubes whatever the internode, suggesting the presence of callose (**Supplementary Figures 5C, D**) and a potential alteration of the transversal elaborate sap conduction.

## Discussion

Studies on Botryosphaeria dieback were already performed at multiple levels using i) *in vitro* grapevine models to better characterize phytotoxic activity of fungal metabolites (Ramírez-Suero et al., 2014; Bénard-Gellon et al., 2015; Trotel-Aziz et al., 2022); ii) greenhouse cuttings and artificial inoculation to understand in-depth grapevine-pathogens interactions (Amponsah et al., 2012; Czemmel et al., 2015; Labois et al., 2020; Obrador-Sanchez and Hernandez-Martinez, 2020) and defense reactions (Urbez-Torres et al., 2010; Lambert et al., 2012; Guan et al., 2016); and iii) in naturally infected vineyards to better characterize the pathogenic species, the response of different cultivars (Larignon et al., 2001; Spagnolo et al., 2014a) and studying the impact of environmental factors on symptom expression (Van Niekerk et al., 2011). If it is now obvious that Botryosphaeriaceae species impact the plant physiology in both wood (Labois et al., 2020) and leaves (Czemmel et al., 2015), but, the stem has so far been less studied (Spagnolo et al., 2014b). Therefore, the main objective of this work was to better characterize the triggering and expression of Botryosphaeria dieback in the stem of plants from vineyard during three stages: the pre-symptomatic (at flowering), the early expression of symptoms (at cluster closure) and the severe symptomatic stage with leaf drop (at late veraison).

### Botryosphaeriaceae infection and vascular obstruction in the vine stem during the expression of leaf symptoms

Our results were suggesting that stems were contaminated annually by Botryosphaeriaceae through the bark from the visible inflorescence stage, possibly by lenticels or buds. We have observed that the expression of leaf symptoms was related to the number of vessels obstructed (by tylosis or gummosis). This number significantly increased from T1 (asymptomatic phase) to T3 (leaf drop), suggesting a potential threshold of obstruction to exceed (25 to 30%) that leads to the expression of symptoms (leaf drop). This observation is therefore different for Esca disease, where the disease severity was not significantly related to the non-functional vessels due to tyloses and gels in leaves (Bortolami et al., 2019). We also observed that the distribution of obstructed vessels in the circumference of internodes seemed relatively random at pre-symptomatic and symptomatic stages. The obstruction affected both the unilateral (in lateral position) and bilateral (in ventral and dorsal positions) vessels of the shoot orthostics (Fournioux and Bessis, 1979) could reduce water supply to leaves, and then lead to the leaf drop process.

### Molecular changes in the vine stem during the expression of leaf symptoms

Genes related to carbohydrate metabolism, defense responses, and secondary meristems activity (vascular cambium and phellogen) were strongly regulated in stems from D plants than from control plants. Of interest, genes associated with the activity of secondary meristems were often down-regulated during symptomatic phase, suggesting physiological and structural responses of the grapevine to the fungal colonization. Gene modulation was less intense at the pre-symptomatic stage than at symptomatic phase, but always different between samples from C and D plants. At T1, defense-related genes were up-regulated in both P and X tissues, suggesting that vines responded to the fungal infection. Moreover, we also reported that *Cal-S7* gene, known to be specific for callose deposition in P (Xie et al., 2011), was induced only in P tissues, as already reported in other woody plants in response to P-vascular agents (Granato et al., 2019).

Similar patterns between P and X samples were observed at symptomatic phase for genes related to carbohydrate metabolism and defense. Targeted defense responses (PR-proteins, superoxide dismutase) were shown to be altered in canes after artificial infections by Botryosphaeriaceae (Spagnolo et al., 2014a; Reis et al., 2016). Interestingly, the down-regulation of *SUC27* at T2 was described as related to embolism formation in X (Chitarra et al., 2014) and water stress (Perrone et al., 2012). Moreover, Chitarra et al. (2014) suggested that, upon X embolism, the main provision of sugars to vessel-associated cells derived from starch breakdown and not from P unloading. This is consistent with our anatomical study where an embolism of the vessels (tylosis) and a strong reduction in amyloplasts were observed. Furthermore, at T2, the expression of genes related to secondary meristems was strongly reduced in P and X samples. The down-regulation of the *APL* gene, required for P differentiation (Furuta et al., 2014; Kalmbach and Helariutta, 2019), could be related with the alteration/disorganization of conductive P observed in diseased canes. The down-regulation of *CDKB2* (meristem regulator; Andersen et al., 2008), *MOL* (required for the formation of secondary vascular tissue in fascicular and interfascicular region; Agusti et al., 2011) and *WOX* (required for auxin-dependent stimulation of cambium activity; Suer et al., 2011) genes could be related with the weakening of the cambium in diseased canes. Finally, the down-regulation of the *SHR* gene (required for phellogen activity; Miguel et al., 2016) was consistent with the lack or the alteration of cane maturation (i.e. “aoûtement”) observed in diseased vines.

### Metabolic changes in the vine stem during the expression of leaf symptoms

The metabolite fingerprints of C and D tissues were similar at the pre-symptomatic stage. Interestingly, glycosylsalicylate was already accumulated in P of declining vines. Ratzinger et al. (2009) also reported an accumulation of SA-glucoside, but in the X sap of shoots, after infection with *Verticillium longisporum*. During symptom occurrence, the stilbenes *cis-* and *trans-*resveratrol and piceid were more accumulated in the X tissues of D samples than of C samples. This result was consistent with the overexpression of *PAL* and *STS* genes in our targeted gene expression analysis. The presence of grapevine phytoalexins (Chong et al., 2009; Loupit et al., 2023) was previously reported in various parts of vines infected by Botryosphaeria dieback (Labois et al., 2020), but also in the green stems of Tempranillo cuttings infected by *N. parvum* and *D. seriata* (Reis et al., 2016), in the wood of Cabernet Sauvignon cuttings infected by *N. parvum* (Massonnet et al., 2018), in Merlot cuttings inoculated by *D. seriata* and *N. parvum* (Lambert et al., 2012), and in the brown stripe area of trunks (Spagnolo et al., 2014a; Lemaître-Guillier et al., 2020). Stilbene production can be induced by proteins secreted by fungi associated to Botryosphaeria dieback (Stempien et al., 2017). Using *in vitro* agar plate assays, Lambert et al. (2012) have shown that Botryosphaeriaceae are very susceptible to stilbenes, but with resveratrol and piceid slightly active.

For both P and X tissues, 1-kestose, a fructan constituted by 3 frutosylfructose units, was similarly accumulated in C and D plants at T1. But, its concentration in D plants was null or weak at T2 and T3, suggesting its concentration was related to disease symptom expression. 1-kestose is known to be produced by several plants such as Banana (Agopian et al., 2008), Asparagus (Forsythe et al., 1990), and grapevine (Dos Santos Lima et al., 2019), and was also described in human to make the resident bifidobacterial more vigorous in the intestinal flora (Hidaka et al., 1986). Mannitol is a polyol commonly found in plants and fungi. In plants, mannitol is as carbon stockpiling compound, an osmolyte, a store of reducing power, and an oxygen radical quencher (for review, see Meena et al., 2015). It is also translocated to source organs when the sucrose pool is drained (Davis and Loescher, 1990), suggesting it can be found in P tissue. In fungi, mannitol has important biological functions under stress conditions, such as the regulation of osmotic pressure and removal of reactive oxygen species (ROS) (Meena et al., 2015). In Arabidopsis/*Alternaria alternata* interaction, the fungus secretes mannitol to quench the plant defense-related ROS production whereas the plant produces mannitol dehydrogenase to catabolize mannitol and preserve this defense event (Jennings et al., 1998). The concentration of mannitol was higher in P and X tissues of D plants than of C plants at T2, suggesting a production by both the grapevine (caused by the fungal infection) and the pathogenic fungus (to quench plant ROS).

### A structural and functional shift in P and X tissues already during the pre-symptomatic stage

We have described structural changes and a dysfunction in both P and X tissues in the stem of internodes of D plants before the shift from the pre-symptomatic to the symptomatic phase.

For the first time, we have shown many alterations in stems before the onset of symptoms, including the obstruction of X vessels (by tylosis and gums) and starch depletion, two processes already mentioned for a trunk dieback (Czemmel et al., 2015). In the pre-symptomatic stage, we have also revealed the simultaneous presence of Botryosphaeriaceae with the secondary P that is disorganized, and appeared collapsed and non-functional regarding callose deposits that could obstruct sieve plates. Callose, a β-1,3-glucan, plugs P sieve tubes during winter and protects vines from cold temperatures (Esau, 1948). Callose is also a defense barrier against pathogens, prevents disease development, and accumulates in wound tissues, becoming a boundary between damaged zone and healing tissue (Taiz and Zeiger, 2002). Callose deposition in the secondary P of woody grape tissue has been studied in relation to seasonal vine development (Aloni and Peterson, 1991; Aloni et al., 1991), but rarely in response to pathogen invasion. Close alterations, with sieve tubes appearing not collapsed, have been described in response to wounding and crown gall in grapevine (Creasap et al., 2005).

Our study also revealed the alteration in the functioning and the establishment of secondary meristems. In diseased vines, the vascular cambium has been able to generate both secondary P and X. However, the vascular meristem appeared collapsed and therefore weakened, in vines suffering from dieback. In addition, we observed that the formation of the periderm is random in diseased vines. In half the cases, the phellogen was not differentiated in a peripheral layer of secondary P in stems. This disorganization is classically reported in other declines of the vine, particularly Pierce’s disease (Stevenson et al., 2005). In the meantime, the defect in the establishment of the periderm and the lack of amyloplasts in the decline vine stems reflected a defect in stem maturation, similarly to the grapevine Pierce’s disease (Stevenson et al., 2005).

## Conclusion

**Figure 9:**
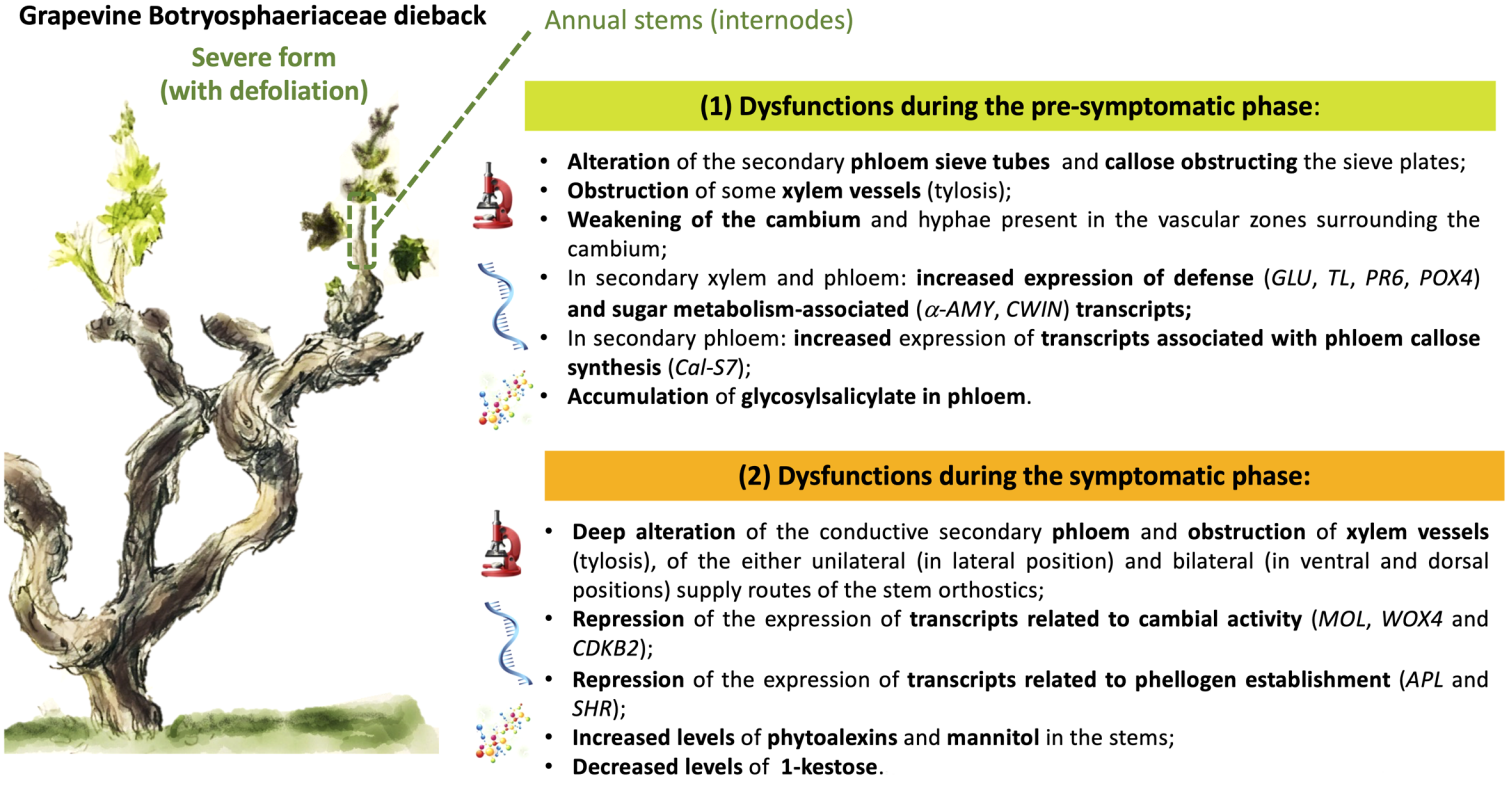
Graphical summary of the main results acquired, reporting the dysfunctions observed during the pre-symptomatic and symptomatic phases of the severe form (leaf drop) of Botryosphaeria dieback in grapevine.

In our study, we combined different approaches to evaluate anatomical, developmental and functional changes related to the presence of Botryosphaeriaceae, both in symptomatic and asymptomatic vine. We have reported structural and molecular modulations from the pre-symptomatic phase T1 (**Figure 9**), with a probable annual contamination of stems through the bark. We have highlighted anatomical and functional disorders in X and P tissues from internodes of the base of stems that may explain symptoms (leaf drop) observed for the severe form of this dieback. At the pre-symptomatic stage (T1), targeted gene expression related to plant defense, sugar metabolism and callose synthesis was consistent to microscopic observations, and highlighted for the first-time P dysfunctions in response to Botryosphaeriaceae. During the symptomatic stage, from early (T2) to leaf drop (T3), a failure to establish the cane maturation (i.e., “aoûtement”) was observed for declining vines and was consistent with (i) the down-regulation of genes related to sucrose signalization, cambial activity and establishment of phellogen, and with (ii) differential accumulation of some metabolites. These plant descriptors of the vine holobiont, involved in plant immunity or wood quality, could be considered as bioindicators of the dysfunctions associated with the severe form of this dieback. Among metabolites, the role of 1-kestose as other ones (i.e., glycosylsalicylate and fructan) in the microbial equilibrium and immunity of vine now deserves to be investigated. In addition, there is a growing interest in understanding the interactions between plants and their microbiome (Pinto et al., 2014; ZarraonaIndia and Gilbert, 2015; Belda et al., 2017; Bettenfeld et al., 2020), especially when related to plant health (Bettenfeld et al., 2022). Thus, in order to have a global view of the vine holobiont dysfunction it would be interesting to evaluate the modulation of the microbial component (i.e., bacteria, fungi and viruses) of the stems associated with the foliar expression of this dieback.

## Data availability statement

The raw data supporting the conclusions of this article will be made available by the authors, without undue reservation.

## Author contributions

FM: Conceptualization, Formal analysis, Investigation, Methodology, Software, Writing – review & editing. LJ: Conceptualization, Formal analysis, Investigation, Methodology, Visualization, Writing – review & editing. PL: Conceptualization, Investigation, Methodology, Writing – review & editing. GC: Data curation, Formal analysis, Investigation, Methodology, Writing – review & editing. CC: Investigation, Methodology, Writing – review & editing. EN: Investigation, Methodology, Software, Writing – review & editing. P-EC: Data curation, Funding acquisition, Project administration, Resources, Validation, Writing – review & editing. FF: Conceptualization, Funding acquisition, Resources, Supervision, Writing – review & editing. MA: Conceptualization, Formal analysis, Funding acquisition, Investigation, Resources, Supervision, Writing – review & editing. ST: Writing – original draft, Writing – review & editing, Conceptualization, Data curation, Formal analysis, Funding acquisition, Investigation, Methodology, Project administration, Resources, Supervision, Validation.

## Supplementary material

Supplementary Figure 1 Presentation of the sequence of samples and samplings carried out during this study. For Botryosphaeria detection by Pasteurian method, six sampling times were analyzed (top of the figure, in orange). For other analyses, the vines sampled on the plot showed 2 distinct health conditions: C (control), D (diseased, severe form). Note that samples “D” of time sample 1 provide from vines that were noted expressive in 2017 (sample collected before symptoms expression). **(A)** For each of the 5 vines identified by modality **(C, D)**, 2 shoots were taken. One was reserved for anatomical characterization and the other one was used for transcriptomic and metabolomic analyzes. **(B)** the samples used for anatomical characterization were sectioned from the base at the 5th or 6th internode and then stored at 4°C until use. **(C)** for the samples planned for the transcriptomic and metabolomic analyzes, fragments of the 3 basal internodes were sectioned, the woody (xylem) and “Phloem/bark” parts were then immediately separated and frozen in liquid nitrogen. The samples were then stored at -80°C until use.

Supplementary Figure 2 Example of illustration of the spatial distribution (canes 1 to 5) of obstructed vessels at the circumference of the stems and along the dorsal/ventral and lateral axes. The data reflect observations made in the second internode. Each layer obtained for each stem taken in a same modality (C or D) presents a different color: cyan for stem 1, blue for stem 2, orange for stem 3, red for stem 4 and black for stem 5. In the case of vines affected by Botryosphaeria dieback (diseased), the T1 (pre-symptomatic) and T3 (symptomatic) times are compared.

Supplementary Figure 3 Hierarchical Cluster Analysis (HCA) analysis of the whole GC-MS data set. “Tissue” corresponds to phloem (P) or xylem (X); “Time” corresponds to sampling time, “1”: before symptom expression (T1), “2”: onset of symptom expression (T2) and “3”: full symptom expression (T3); “Expression” corresponds to Control (C) and Diseased (D) samples; class corresponds to the internode position (1, 2 and 3 from the base of the stem).

Supplementary Figure 4 Focus on structural observations and hyphae localization in grapevine stems harvested at T2 (symptomatic stage) from diseased plants. Observations were made in the internode of the base of the stems (internode 1), both at the level of the secondary phloem **(A, B)** and of the secondary xylem **(C, E)**. The presence of hyphae is indicated by arrows.

Supplementary Figure 5 Evaluation of the presence of callose in the vascular secondary liber of control **(A, B)** and diseased **(C, D)** plants, detected after aniline blue staining. The staining is specific for β-1,3-glucan and stains the callose in intense blue.

Supplementary File 1 Climatic data (temperatures and rainfall) collected between 2010 and 2021.

Supplementary Table 1 Primer sets used to detect Botryosphaeriaceae in grapevine stems.

Supplementary Table 2 Sequences of the primers derived from *Vitis vinifera* and used for RT-q-PCR.

Supplementary Table 3 List of the metabolites differently accumulated in the internode phloem of control and diseased grapevines. GC-analysis was performed on phloem tissues collected at different times (T1, from pre-symptomatic stems; T2 and T3 from symptomatic ones). “Pvalue”: p value corresponding to the T-test, “FC”: fold change C/D, “id”: identification number indicated on Volcano plots, “C > D”: metabolites more accumulated in control samples than in diseased ones. “D > C”: metabolites more accumulated in diseased samples than in control ones.

Supplementary Table 4 List of the metabolites differently accumulated in the internode xylem of control and diseased grapevines. GC-analysis was performed on samples collected at different times (T1, from pre-symptomatic stems; T2 and T3 from symptomatic ones). “Pvalue”: p value corresponding to the T-test, “FC”: fold change C/D, “id”: identification number indicated on Volcano plots, “C > D”: metabolite more accumulated in control samples than in D ones. “D > C”: metabolite more accumulated in diseased samples than in control ones.
