# Supplementary figures and images for: Physiological and developmental disturbances caused by Botryosphaeria dieback in the annual stems of grapevine
Source: Front Plant Sci. 2024 Apr 23;15:1394821. doi: 10.3389/fpls.2024.1394821 (PMC11074360; doi:10.3389/fpls.2024.1394821)

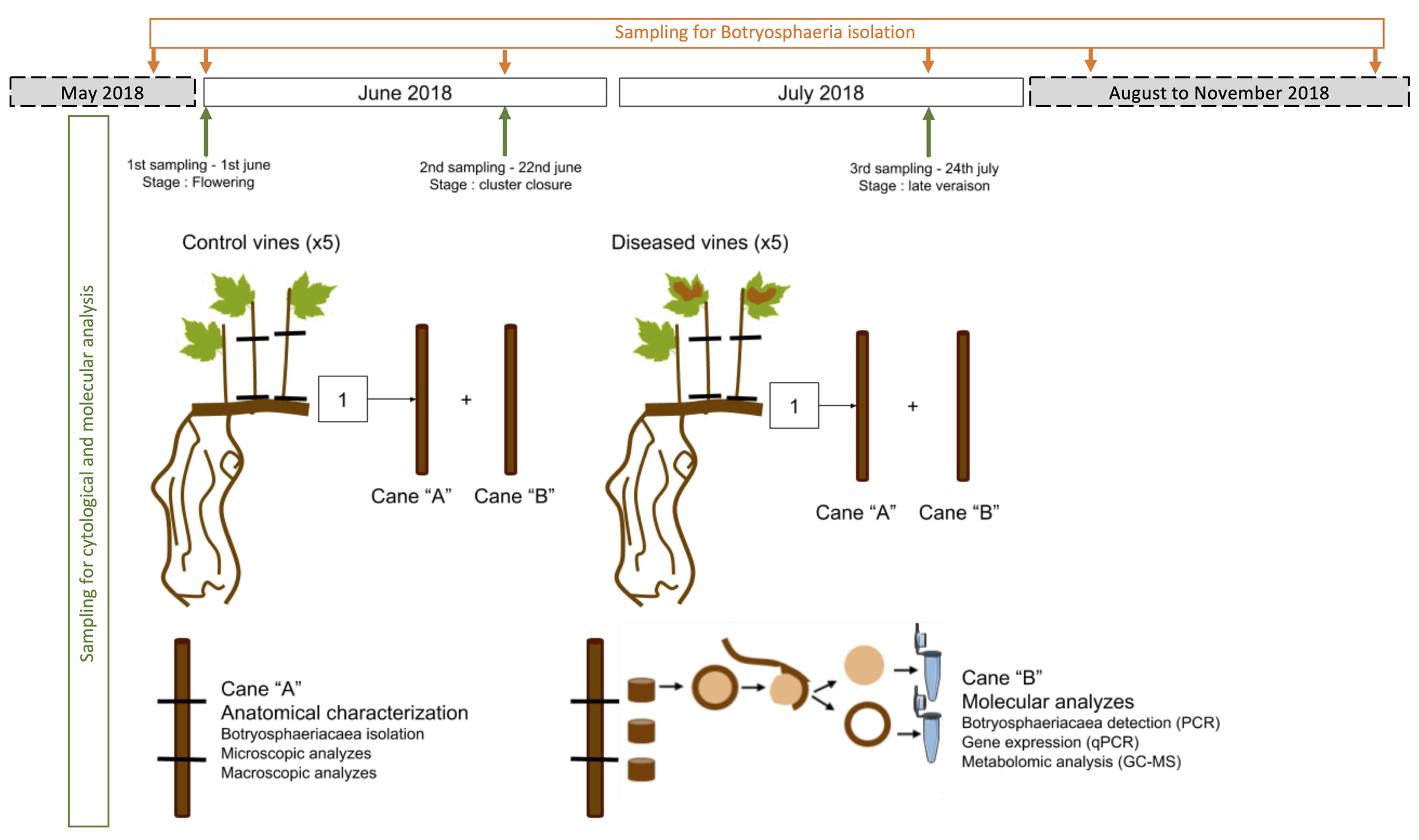

Supplement: Supplementary Figure 1 — Presentation of the sequence of samples and samplings carried out during this study. For Botryosphaeria detection by Pasteurian method, six sampling times were analyzed (top of the figure, in orange). For other analyses, the vines sampled on the plot showed 2 distinct health conditions: C (control), D (diseased, severe form). Note that samples “D” of time sample 1 provide from vines that were noted expressive in 2017 (sample collected before symptoms expression). (A) For each of the 5 vines identified by modality (C, D), 2 shoots were taken. One was reserved for anatomical characterization and the other one was used for transcriptomic and metabolomic analyzes. (B) the samples used for anatomical characterization were sectioned from the base at the 5th or 6th internode and then stored at 4°C until use. (C) for the samples planned for the transcriptomic and metabolomic analyzes, fragments of the 3 basal internodes were sectioned, the woody (xylem) and “Phloem/bark” parts were then immediately separated and frozen in liquid nitrogen. The samples were then stored at -80°C until use. [file Image_1.tif]

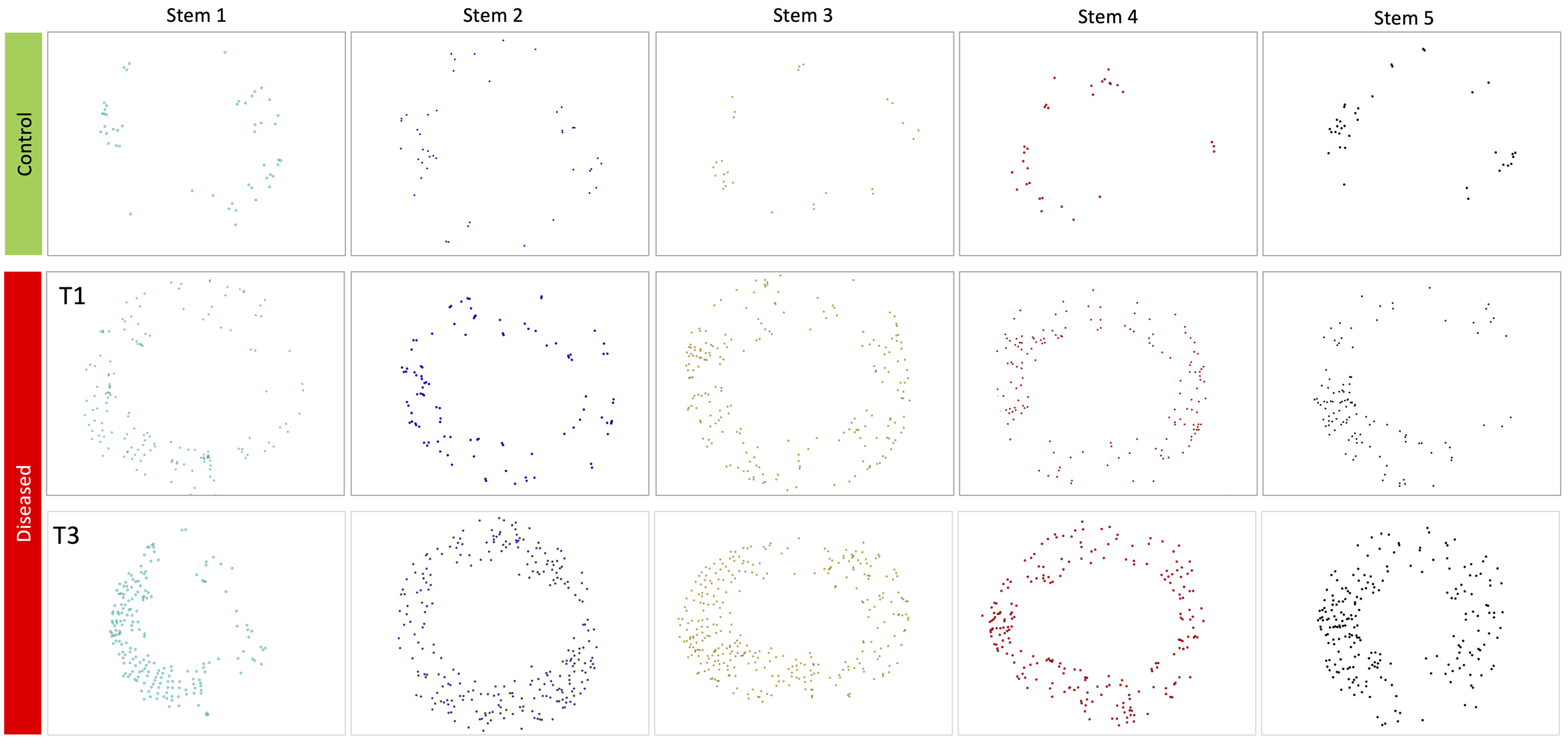

Supplement: Supplementary Figure 2 — Example of illustration of the spatial distribution (canes 1 to 5) of obstructed vessels at the circumference of the stems and along the dorsal/ventral and lateral axes. The data reflect observations made in the second internode. Each layer obtained for each stem taken in a same modality (C or D) presents a different color: cyan for stem 1, blue for stem 2, orange for stem 3, red for stem 4 and black for stem 5. In the case of vines affected by Botryosphaeria dieback (diseased), the T1 (pre-symptomatic) and T3 (symptomatic) times are compared. [file Image_2.tif]

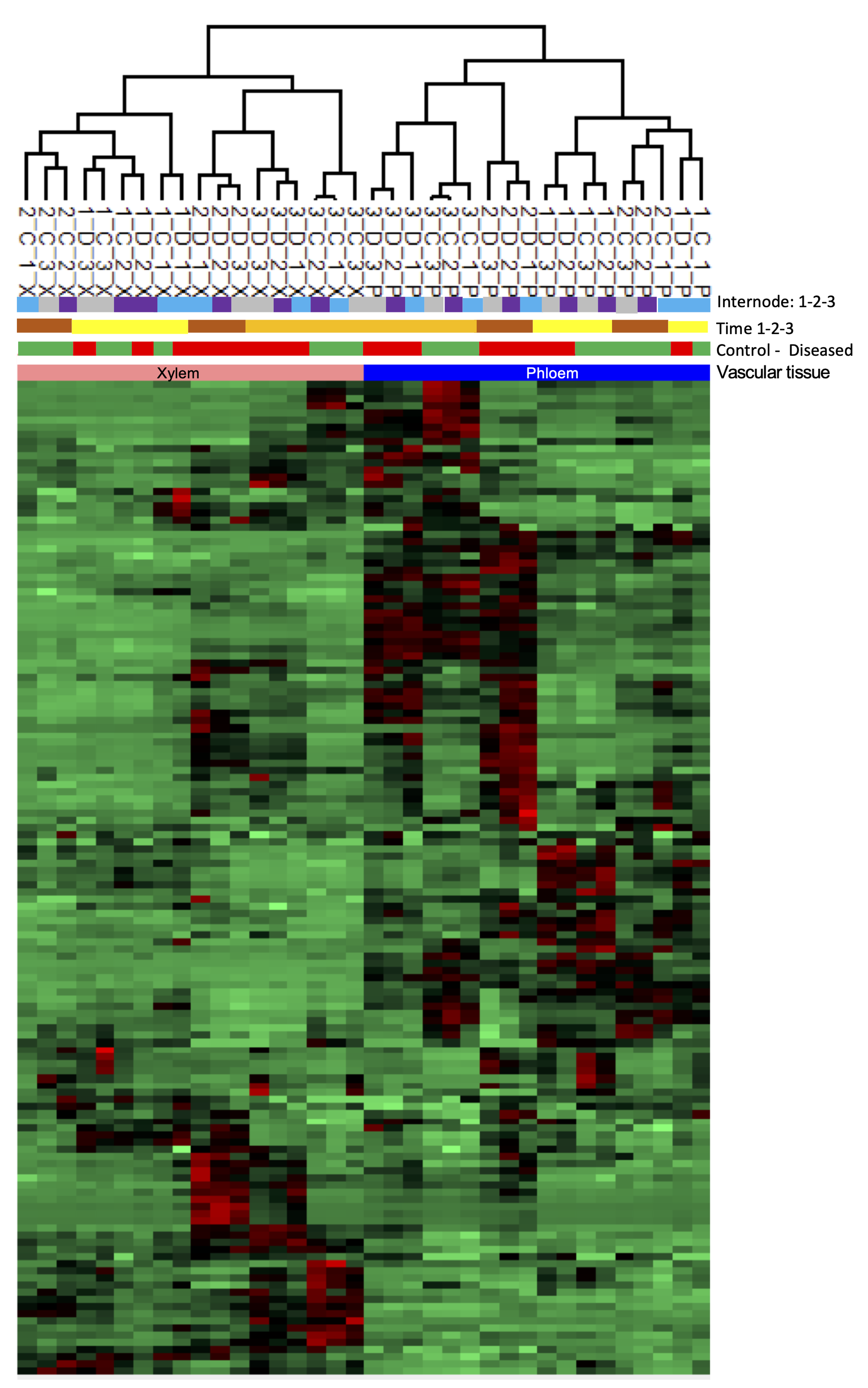

Supplement: Supplementary Figure 3 — Hierarchical Cluster Analysis (HCA) analysis of the whole GC-MS data set. “Tissue” corresponds to phloem (P) or xylem (X); “Time” corresponds to sampling time, “1”: before symptom expression (T1), “2”: onset of symptom expression (T2) and “3”: full symptom expression (T3); “Expression” corresponds to Control (C) and Diseased (D) samples; class corresponds to the internode position (1, 2 and 3 from the base of the stem). [file Image_3.tif]

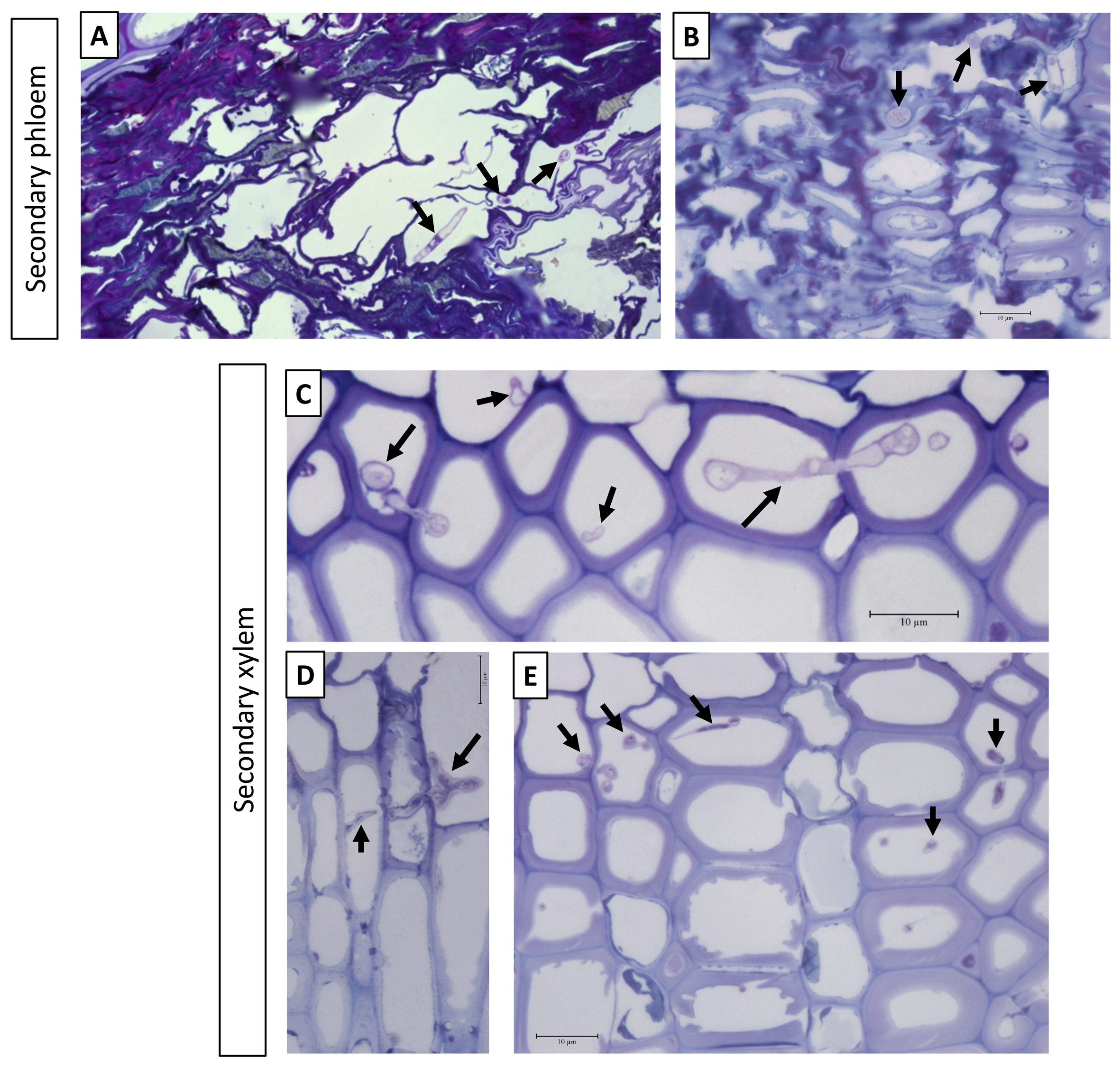

Supplement: Supplementary Figure 4 — Focus on structural observations and hyphae localization in grapevine stems harvested at T2 (symptomatic stage) from diseased plants. Observations were made in the internode of the base of the stems (internode 1), both at the level of the secondary phloem (A, B) and of the secondary xylem (C, E). The presence of hyphae is indicated by arrows. [file Image_4.tif]

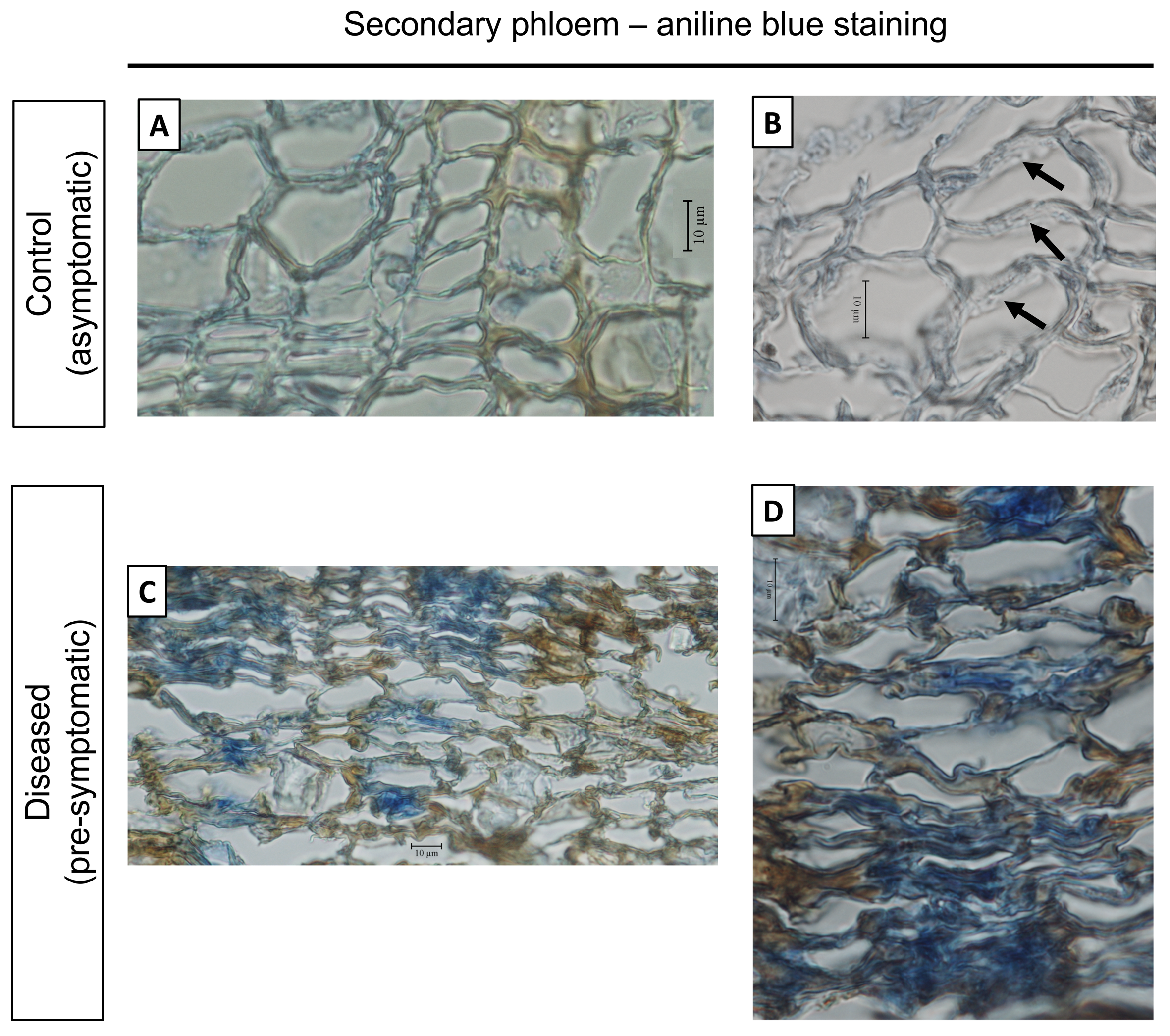

Supplement: Supplementary Figure 5 — Evaluation of the presence of callose in the vascular secondary liber of control (A, B) and diseased (C, D) plants, detected after aniline blue staining. The staining is specific for β-1,3-glucan and stains the callose in intense blue. [file Image_5.tif]
