# Supplementary material for: Physiological and developmental disturbances caused by Botryosphaeria dieback in the annual stems of grapevine
Source: Front Plant Sci. 2024 Apr 23;15:1394821. doi: 10.3389/fpls.2024.1394821 (PMC11074360; doi:10.3389/fpls.2024.1394821)
Supplement: Supplementary Table 1 — Primer sets used to detect Botryosphaeriaceae in grapevine stems. [file Table_1.docx]

**Supplemental Tables.**

**Supplemental Table 1**: Primer sets used to detect Botryosphaeriaceae in grapevine stems.

| Primer pairs | Primer sequences (5’→ 3’) | Reference |
| --- | --- | --- |
| ITS1-F | CTTGGTCATTTAGAGGAAGTAA | Udayanga et al. (2012) |
| ITS4 | TCCTCCGCTTATTGATATGC |  |
| BOT_100F | AAACTCCAGTCAGTRAAC | Ridgway et al. (2011) |
| BOT_472R | TCCGAGGTCAMCCTTGAG |  |

**Supplemental Table 2**: Sequences of the primers derived from *Vitis vinifera* and used for RT-q-PCR.

| Gene Functions | Gene​ Names | | Gene bank accession number​ | Forward primers (5’>3’)​ | Reverse primers (5’> 3’)​ | Reference​ |  |
| --- | --- | --- | --- | --- | --- | --- | --- |
|  |  |  |  |  |  |  |  |
| Housekeeping | 60S ribosomal protein L18 | 60S | XM_002270599.3 | GATATAACAGGGAATACAGCAC | ATCTACCTCAAGCTCCAGTC | Reis et al., 2016 |  |
|  | Elongation factor 1-alpha | EF1-α | GU585871 | AACCAAAATATCCGGAGTAAAAGA​ | GAACTGGGTGCTTGATAGGC​ | Spagnolo et al., 2014a |  |
| Defense | Callose synthase 7 | Cal-S7 | XM_010658818.2 | AGAGGATACGGTGGGTGGAA | GTTTGCACAGCTTCTCCACG | This study |  |
|  | β-1,3 Glucanase | GLU | AF 239617 | TCAATGGCTGCAATGGTGC | CGGTCGATGTTGCGAGATTTA | Reis et al., 2016 |  |
|  | Glutathione-S-transferase 5 | GST5 | XM_ 002277883 | GCAGAAGCTGCCAGTGATTGGA | GCAGAAGCTGCCAGTGATTGGA | Reis et al., 2016 |  |
|  | Peroxidase 4 | POX4 | XM_002269882 | AACATCCCCCCTCCCACTT | TGCATCTCGCTTGGCCTATT | Spagnolo et al., 2014a |  |
|  | Phenylalanine ammonia lyase | PAL | X75967 | TCCTCCCGGAAAACAGCTG | TCCTCCAAATGCCTCAAATCA | Spagnolo et al., 2017 |  |
|  | Serine protease inhibitor | PR6 | AY156047.1 | AGGGAACAATCGTTACCCAAG | CCGATGGTAGGGACACTGAT | Reis et al., 2016 |  |
|  | Stillbene synthase | STS | X76892 | AGGAAGCAGCATTGAAGGCTC | TGCACCAGGCATTTCTACACC |  |  |
|  | Thaumatin-like 1 | TL1 | AF532965 | GGCCATAGGCACATTAAATCCATC | CCTAACACCTTAGCCGAATTCGC | Spagnolo et al., 2017 |  |
|  | Wall Are Thin 1 | WAT1 | XM_002280026.4 | GAAGAAGTTTGCCGCCAAGG | GGGTTAGGGTTTGCTGGTCA | This study |  |
| Detoxification and stress tolerance | Epoxide hydrolase 2 | epoxH2 | XM_002270484 | TCTGGATTCCGAACTGCATTG | ACCCATGATTAGCAGCATTGG | Spagnolo et al., 2014a |  |
|  | Superoxide dismutase | SOD | AF056622.1 | GTGGACCTAATGCAGTGATTGGA | TGCCAGTGGTAAGGCTAAGTTCA | Spagnolo et al., 2017 |  |
| Phellogen | Myb family transcription factor APL | APL | XM_010649920.2 | TGGGTCGCAACATAAATGAGAA | CAGTCTCCGTTTTTGCCTGT | This study |  |
|  | Epidermal growth factor binding protein | EBP1 | XM_002262950.4 | TCCCAGTCCAGAGACCAGAG | TCCCATCACCTGTGCTTGTG | This study |  |
|  | Plasma membrane ATPase4 | HA3 | XM_002267465.3 | GACTGCTGTTAGTCACCGCT | CCCCAGCCCATTCCTTTGAT | This study |  |
|  | Phytosulfokine receptor 1 | PSKR1 | XM_002273150.3 | GCCCACTTGGCTGATTTTGG | CTTGGCCATACTCAGGTGGG | This study |  |
|  | Phytosulfokine receptor 2 | PSKR2 | XM_002282552.3 | AGATCCAAGGCAGAGACCCT | GTTACACGCCCTCCTTTCCA | This study |  |
|  | Protein short-root | SHROOT | XM_019218197.1 | CCCTAAGTTCAGTGGCGGAG | GCAAAGTGGGCCATTGAGTG | This study |  |
| Sucrose metabolism and signalisation | Alpha amylase | α-AMY | XM_002285177.3 | AGCTTGTGGACTGGGTGAAA | GAGGCCCTCCATTTGAGTCC | Sawicki et al., 2015 |  |
|  | Beta amylase | β-AMY | XM_002265662.3 | TACCATACACTATCACACCC | CATAATCCAGCCTTATCAAACC |  |  |
|  | Cell Wall apoplastic Invertase | CWINV | NM_001281279.1 | TATTGACGGTGAAGCCCACT | AAAGCCTGGCTCTTCACTCA | Santi et al., 2013 |  |
|  | Sucrose transporter-like | SUC27 | NM_001281141.3 | TGGGTGCTTCTCCTTGTGAC | CCCAGATCGTACAGCCTTCC |  |  |
| Vascular cambium | Cyclin-dependant kinase B2-1 | CDKB2 | XM_002276114.3 | CTGGAGAAGGTAGGGGAAGG | AACATGCGAAGCAAGGAGAC | Vergara et al., 2017 |  |
|  | Ethylene response factor 5 | ERF5 | XM_002281918.3 | TGACGACGAATGCTCTACCTC | CGGAGGAACTGAGATGCACA | This study |  |
|  | More lateral growth | MOL | XM_002264494.4 | CTGGGGAGCAAAAGCAAAGAC | GGAAGCCCGCTAAAAGAAACC | This study |  |
|  | Wulschel-related homeobox 4 | WOX4 | XM_002284891.3 | CACTCCCAATGCACAGCAAA | CTGCTTCTGTCTCTCCCGTG | This study |  |

**Supplemental references cited in this table:**

- Santi, S., De Marco, F., Polizzotto, R., Grisan, S., and Musetti., R. (2013). Recovery from stolbur disease in grapevine involves changes in sugar transport and metabolism. Front. Plant Sci. 4. doi: 10.3389/fpls.2013.00171
- Sawicki, M., Jacquens, L., Baillieul, F., Clément, C., Vaillant-Gaveau, N., and Jacquard, C. (2015). Distinct regulation in inflorescence carbohydrate metabolism according to grapevine cultivars during floral development. Physiol. Plant 154, 447–467. doi: 10.1111/ppl.12321
- Spagnolo, A., Mondello, V., Larignon, P., Villaume, S., Rabenoelina, F., Clément, C., et al. (2017). Defense Responses in Grapevine (cv. Mourvèdre) after Inoculation with the Botryosphaeria Dieback Pathogens *Neofusicoccum parvum* and *Diplodia seriata* and Their Relationship with Flowering. Int. J. Mol. Sci. 18, 393. doi: 10.3390/ijms18020393
- Vergara, R., Noriega, X., Aravena, K., Prieto, H., and Pérez, F. J. (2017). ABA represses the expression of cell cycle genes and may modulate the development of endodormancy in grapevine buds. Front. Plant Sci. 8. doi: 10.3389/fpls.2017.00812

**Supplemental Table 3**: List of the metabolites differently accumulated in the internode phloem of control and diseased grapevines. GC-analysis was performed on phloem tissues collected at different times (T1, from pre-symptomatic stems; T2 and T3 from symptomatic ones). “Pvalue”: p value corresponding to the T-test, “FC”: fold change C/D, “id”: identification number indicated on Volcano plots, “C > D”: metabolites more accumulated in control samples than in diseased ones. “D > C”: metabolites more accumulated in diseased samples than in control ones.

| P-T1 | **metabolites** | pval | mlog10pval | FC | log2FC | id |
| --- | --- | --- | --- | --- | --- | --- |
| D > C | Glycosylsalicylate | 0.00288901 | 2.539251 | 0.4291085 | -1.220586 | 64 |
|  |  |  |  |  |  |  |
| P-T2 | **metabolites** | pval | mlog10pval | FC | log2FC | id |
| C > D | 1-Kestose | 0.01174703 | 1.930072 | 78.644128 | 6.297267 | 71 |
|  | Digalactosylglycerol | 0.00474459 | 2.323801 | 9.409446 | 3.23411 | 31 |
|  | Threonate | 0.00270094 | 2.568484 | 4.907497 | 2.294987 | 110 |
|  | Citrate | 0.01988285 | 1.701521 | 4.120093 | 2.042677 | 26 |
|  | Cysteine | 0.01888525 | 1.723877 | 4.094303 | 2.033618 | 29 |
|  | Raffinose | 0.04782581 | 1.320338 | 3.951711 | 1.982477 | 95 |
|  | Glucose | 0.00202898 | 2.692723 | 3.436491 | 1.780936 | 59 |
|  | Galactinol | 0.01969318 | 1.705684 | 3.176127 | 1.667269 | 47 |
|  | U2025.2.204 | 0.00835188 | 2.078216 | 2.88772 | 1.529931 | 176 |
|  | Pantothenate | 0.01259457 | 1.899817 | 2.662003 | 1.412512 | 80 |
|  | U1991.0.333 | 0.02557027 | 1.592265 | 2.487483 | 1.314687 | 171 |
|  | Histidine | 0.01265802 | 1.897634 | 2.429686 | 1.28077 | 65 |
|  | U2015.292 | 0.03294326 | 1.482233 | 2.15968 | 1.110818 | 174 |
|  | Sucrose | 0.03372216 | 1.472085 | 2.020275 | 1.014552 | 106 |
| D > C | **metabolites** | pval | mlog10pval | FC | log2FC | id |
|  | Mannitol | 9.18E-04 | 3.037019 | 0.01811206 | -5.786906 | 75 |
|  | Resveratrol.cis | 1.38E-02 | 1.859068 | 0.06101456 | -4.034703 | 96 |
|  | Leucine | 8.78E-03 | 2.056509 | 0.09464977 | -3.401257 | 72 |
|  | Resveratrol.trans | 8.72E-06 | 5.059503 | 0.10462659 | -3.256679 | 97 |
|  | Succinate | 3.68E-03 | 2.43369 | 0.1279104 | -2.966794 | 105 |
|  | Isoleucine | 1.06E-02 | 1.973167 | 0.15658058 | -2.675023 | 68 |
|  | Nicotinate | 2.88E-05 | 4.54063 | 0.15702038 | -2.670976 | 78 |
|  | Threitol | 1.87E-04 | 3.729138 | 0.16583192 | -2.592206 | 109 |
|  | Piceid | 2.60E-03 | 2.585519 | 0.17190739 | -2.540297 | 87 |
|  | Erythritol | 1.72E-03 | 2.764049 | 0.17390307 | -2.523645 | 34 |
|  | Citramalate | 5.43E-05 | 4.264919 | 0.17843387 | -2.486539 | 25 |
|  | U1601.5.292 | 2.43E-03 | 2.615087 | 0.18133552 | -2.463267 | 138 |
|  | Homoserine | 1.57E-03 | 2.804475 | 0.22900242 | -2.126565 | 66 |
|  | Xylose | 3.30E-03 | 2.481789 | 0.26982791 | -1.889888 | 299 |
|  | Valine | 2.48E-02 | 1.606014 | 0.2938595 | -1.766802 | 298 |
|  | Glycosylsalicylate | 1.61E-02 | 1.792881 | 0.29391721 | -1.766518 | 64 |
|  | Arbutin | 2.92E-02 | 1.534589 | 0.29469408 | -1.76271 | 12 |
|  | Malate | 1.75E-02 | 1.757364 | 0.30686913 | -1.704305 | 72 |
|  | Rhamnose | 1.89E-03 | 2.722712 | 0.32230601 | -1.33497 | 98 |
|  | Phytol | 4.90E-03 | 2.309372 | 0.34552723 | -1.533129 | 85 |
|  | Alanine | 5.87E-03 | 2.231171 | 0.37931994 | -1.398513 | 8 |
|  | Maltose | 1.36E-02 | 1.867243 | 0.40672088 | -1.297889 | 74 |
|  | Fructose.6.P | 1.28E-02 | 1.893838 | 0.40790544 | -1.293693 | 43 |
|  | alpha.Tocopherol | 7.52E-03 | 2.123894 | 0.47487368 | -1.074384 | 9 |
|  | Galactosylglycerol.3 | 4.77E-03 | 2.321461 | 0.48289533 | -1.050218 | 51 |
|  | Pyruvate | 3.61E-03 | 2.442539 | 0.48855627 | -1.033403 | 91 |
|  | Catechin | 7.15E-03 | 2.145892 | 0.49036898 | -1.02806 | 23 |
|  | U2106.245 | 2.79E-02 | 1.554764 | 0.49959302 | -1.001175 | 180 |
| P-T3 | **metabolites** | pval | mlog10pval | FC | log2FC | id |
| C > D | 1-Kestose | 1.09E-02 | 1.963671 | 5.665681 | 2.502249 | 71 |
|  | Threonate | 3.16E-03 | 2.499653 | 5.656193 | 2.499831 | 110 |
|  | Phenylalanine | 5.32E-04 | 3.27371 | 4.894586 | 2.291187 | 83 |
|  | Cysteine | 2.02E-03 | 2.694496 | 4.56398 | 2.190293 | 29 |
|  | Digalactosylglycerol | 3.05E-03 | 2.515968 | 3.486246 | 1.801675 | 31 |
|  | Raffinose | 2.54E-02 | 1.59448 | 2.612883 | 1.385643 | 95 |
|  | Glycine | 3.77E-02 | 1.423522 | 2.049232 | 1.035083 | 63 |
|  | Xylose | 2.67E-02 | 1.573565 | 2.04675 | 1.033335 | 299 |
|  | Citrate | 1.22E-05 | 4.912855 | 2.041316 | 1.029499 | 26 |
| D > C | **metabolites** | pval | mlog10pval | FC | log2FC | id |
|  | U2106.245 | 1.40E-03 | 2.855302 | 0.19024881 | -2.394041 | 180 |
|  | Glutamine | 1.12E-02 | 1.949324 | 0.19937394 | -2.326451 | 54 |
|  | Isoleucine | 8.61E-05 | 4.064998 | 0.2287213 | -2.128337 | 68 |
|  | Leucine | 5.11E-05 | 4.291839 | 0.247464 | -2.014709 | 72 |
|  | Valine | 2.30E-03 | 2.638094 | 0.27447282 | -1.865265 | 298 |
|  | Homoserine | 2.68E-02 | 1.571236 | 0.35440991 | -1.496509 | 66 |
|  | U1601.5.292 | 2.60E-02 | 1.584435 | 0.38070752 | -1.393245 | 138 |
|  | Malate | 1.05E-02 | 1.978704 | 0.38098222 | -1.392204 | 72 |
|  | Glycerate | 1.76E-03 | 2.755472 | 0.38763857 | -1.367216 | 61 |
|  | Alanine | 7.37E-03 | 2.132385 | 0.41282651 | -1.276392 | 8 |
|  | Phenylalanine | 2.40E-02 | 1.619428 | 0.44536338 | -1.166945 | 83 |
|  | Galactonate | 2.54E-02 | 1.595691 | 0.46046919 | -1.118823 | 50 |
|  | Succinate | 1.61E-03 | 2.792278 | 0.46537674 | -1.103529 | 105 |

**Supplemental Table 4**: List of the metabolites differently accumulated in the internode xylem of control and diseased grapevines. GC-analysis was performed on samples collected at different times (T1, from pre-symptomatic stems; T2 and T3 from symptomatic ones). “Pvalue”: p value corresponding to the T-test, “FC”: fold change C/D, “id”: identification number indicated on Volcano plots, “C > D”: metabolite more accumulated in control samples than in D ones. “D > C”: metabolite more accumulated in diseased samples than in control ones.

| X-T1 | **metabolites** | pval | mlog10pval | FC | log2FC | id |
| --- | --- | --- | --- | --- | --- | --- |
|  |  |  |  |  |  |  |
| X-T2 | **metabolites** | pval | mlog10pval | FC | log2FC | id |
| C > D | 1-Kestose | 0.00015383 | 3.812963 | 88.146163 | 6.461826 | 16 |
|  | Galactinol | 0.00038243 | 3.41745 | 5.184176 | 2.374115 | 32 |
|  | U2923.9.456 | 0.00774788 | 2.110817 | 4.032548 | 2.011692 | 47 |
|  | Threonine | 0.0103948 | 1.983184 | 3.730837 | 1.899499 | 63 |
|  | Epicatechin | 0.00273763 | 2.562626 | 2.732517 | 1.450231 | 71 |
|  | Sucrose | 0.00207295 | 2.683411 | 2.68036 | 1.422427 | 94 |
|  | Quinate | 0.0012422 | 2.905807 | 2.503472 | 1.32393 | 95 |
|  | Aspartate | 0.02281422 | 1.641794 | 2.430126 | 1.281031 | 106 |
|  | Glycine | 0.01019096 | 1.991785 | 2.411547 | 1.269959 | 112 |
|  | Raffinose | 0.00744624 | 2.128063 | 2.140195 | 1.097743 | 176 |
|  | U2025.2.204 | 0.03967878 | 1.401442 | 2.010805 | 1.007773 | 257 |
|  | Valine | 0.04446304 | 1.352001 | 2.005967 | 1.004298 | 298 |
| D > C | **metabolites** | pval | mlog10pval | FC | log2FC | id |
|  | Resveratrol.trans | 1.42E-03 | 2.84868 | 0.00163061 | -9.260374 | 97 |
|  | Resveratrol.cis | 1.10E-05 | 4.959157 | 0.01423819 | -6.13409 | 96 |
|  | Mannitol | 1.21E-02 | 1.915528 | 0.01839879 | -5.764245 | 75 |
|  | Piceid | 2.27E-03 | 2.64472 | 0.0376614 | -4.73077 | 87 |
|  | Threitol | 1.50E-03 | 2.823851 | 0.05380845 | -4.216023 | 109 |
|  | Erythritol | 1.53E-03 | 2.815032 | 0.05550516 | -4.171234 | 34 |
|  | Nicotinate | 8.74E-04 | 3.05855 | 0.06766373 | -3.885473 | 78 |
|  | Citramalate | 3.82E-06 | 5.418112 | 0.09650006 | -3.373326 | 25 |
|  | U1601.5.292 | 7.90E-04 | 3.102386 | 0.1213748 | -3.042459 | 138 |
|  | Aconitate | 2.03E-02 | 1.693266 | 0.12229997 | -3.031504 | 5 |
|  | Glycerate | 8.74E-03 | 2.058267 | 0.12811364 | -2.964504 | 61 |
|  | Arbutin | 7.85E-05 | 4.104867 | 0.13339683 | -2.906204 | 13 |
|  | Succinate | 1.43E-02 | 1.844046 | 0.14289507 | -2.806972 | 105 |
|  | Galactosylglycerol | 1.77E-02 | 1.753135 | 0.15126028 | -2.724895 | 51 |
|  | Glycosylsalicylate | 1.80E-03 | 2.74549 | 0.18673434 | -2.420941 | 64 |
|  | Malate | 4.22E-04 | 3.374797 | 0.26504117 | -1.915712 | 73 |
|  | U1747.0.394 | 3.93E-03 | 2.405199 | 0.33260328 | -1.588126 | 148 |
|  | U2206.2.299 | 8.71E-03 | 2.060053 | 0.35595658 | -1.490227 | 193 |
|  | Arabinose | 4.13E-02 | 1.384515 | 0.36398462 | -1.458051 | 11 |
|  | Quercetin.3.O.glucuronide | 1.39E-03 | 2.855881 | 0.41527792 | -1.267851 | 93 |
|  | Alanine | 2.46E-02 | 1.609286 | 0.42660865 | -1.229015 | 8 |
|  | Rhamnose | 1.98E-03 | 2.703005 | 0.44385077 | -1.171853 | 98 |
|  | Ribonate | 3.49E-02 | 1.457533 | 0.44681326 | -1.162256 | 99 |
|  | Tartrate | 6.29E-03 | 2.201256 | 0.45514255 | -1.13561 | 108 |
|  | Fructose.6.P | 6.93E-03 | 2.159363 | 0.46274291 | -1.111717 | 43 |
|  | U2375.6.191 | 1.43E-03 | 2.846057 | 0.46751591 | -1.096913 | 202 |
|  | Xylose | 1.84E-02 | 1.735548 | 0.46833384 | -1.094391 | 199 |
| X-T3 | **metabolites** | pval | mlog10pval | FC | log2FC | id |
| C > D | Raffinose | 0.00179697 | 2.74546 | 7.208037 | 2.849606 | 95 |
|  | Digalactosylglycerol | 0.00888866 | 2.051164 | 6.210422 | 2.634691 | 31 |
|  | 1-Kestose | 0.00805069 | 2.094167 | 6.180249 | 2.627665 | 71 |
|  | Ascorbate | 0.04933549 | 1.306841 | 5.992057 | 2.583051 | 15 |
|  | U2923.9.456 | 0.00782508 | 2.106511 | 2.950046 | 1.560737 | 257 |
|  | Sucrose | 0.00176637 | 2.75292 | 2.827284 | 1.499417 | 106 |
|  | Cysteine | 0.02197594 | 1.658053 | 2.514387 | 1.330207 | 29 |
|  | Threonate | 0.01552382 | 1.809002 | 2.413404 | 1.271069 | 110 |
|  | U1991.0.333 | 0.00230693 | 2.636966 | 2.372222 | 1.246239 | 171 |
|  | Quinate | 0.00374109 | 2.427001 | 2.328594 | 1.219459 | 94 |
|  | Pantothenate | 0.00290579 | 2.536736 | 2.11002 | 1.077257 | 80 |
|  |  |  |  |  |  |  |
| D > C | **metabolites** | pval | mlog10pval | FC | log2FC | id |
|  | Resveratrol.trans | 1.29E-02 | 1.888138 | 0.00652657 | -7.25946 | 97 |
|  | Resveratrol.cis | 3.41E-02 | 1.467662 | 0.01626943 | -5.941693 | 96 |
|  | Piceid | 1.15E-02 | 1.940499 | 0.0639276 | -3.967417 | 87 |
|  | Threitol | 1.54E-02 | 1.813851 | 0.07903222 | -3.661415 | 109 |
|  | Erythritol | 3.30E-04 | 3.480851 | 0.08916054 | -3.487451 | 34 |
|  | U1601.5.292 | 4.35E-04 | 3.361816 | 0.12055411 | -3.052247 | 138 |
|  | Succinate | 1.48E-03 | 2.830407 | 0.22241729 | -2.168659 | 105 |
|  | Glycerate | 3.25E-06 | 5.487842 | 0.2264713 | -2.1426 | 61 |
|  | Citramalate | 2.22E-05 | 4.65286 | 0.2860221 | -1.805801 | 25 |
|  | Rhamnose | 3.61E-03 | 2.441984 | 0.30627117 | -1.707119 | 98 |
|  | Glycosylsalicylate | 4.16E-04 | 3.381013 | 0.32490028 | -1.621931 | 64 |
|  | Malate | 2.03E-04 | 3.693128 | 0.32619655 | -1.616187 | 73 |
|  | Arabitol | 2.11E-03 | 2.676186 | 0.36423954 | -1.457041 | 12 |
|  | Arbutin | 4.83E-03 | 2.316289 | 0.40564264 | -1.301719 | 13 |
|  | Galactosylglycerol | 2.44E-02 | 1.612442 | 0.43602104 | -1.19753 | 51 |
|  | Adenosine.5.P | 2.95E-02 | 1.529739 | 0.43611817 | -1.197209 | 7 |
